# Clays as Additives in Ruminant Feed: A Quantitative Meta-Analysis of Their Effects on Enteric Methane, Digestion, Health and Productivity

**DOI:** 10.3390/ani16172646

**Published:** 2026-08-24

**Authors:** Zubaer Hosen, Md. Rashidul Islam, Ravi Naidu, Bhabananda Biswas

**Affiliations:** 1 Global Centre for Environmental Remediation, The University of Newcastle, Callaghan, NSW 2308, Australia; mdrashidul.islam@newcastle.edu.au (M.R.I.); ravi.naidu@newcastle.edu.au (R.N.); 2 crc for Contamination Assessment and Remediation of the Environment (crcCARE), University Drive, Callaghan, NSW 2308, Australia

**Keywords:** enteric fermentation, feed additives, livestock nutrition, meta-regression, sustainable ruminant production, systematic review

## Abstract

Clays are increasingly incorporated as feed additives for ruminants, partly inspired by animals’ natural geophagic behaviour. However, their effects on rumen function and productivity remain inconsistent in the literature. This meta-analysis synthesised 39 controlled studies (73 treatment means) to quantitatively evaluate the effects of clay supplementation on fermentation, digestibility, metabolic health, and productivity. Clay supplementation improved rumen fermentation efficiency, as indicated by reductions in methane emissions (−20.45%; *p* < 0.001) and ammonia-N (−11.51%; *p* = 0.033), along with an increase in propionate production (+4.35%; *p* = 0.046). These findings are consistent with proposed mechanisms not directly evaluated in this meta-analysis. Total volatile fatty acid concentration (+3.25%; *p* = 0.20) and rumen pH (+0.25%; *p* = 0.75) were not significantly altered by clay supplementation. Digestibility responses were moderately improved, particularly for neutral detergent fibre (+7.32%; *p* = 0.011) and organic matter (+3.06%; *p* < 0.001). These improvements may reflect enhanced microbial colonisation and substrate utilisation. Blood urea nitrogen decreased (−5.64%; *p* = 0.002) with minimal systemic effects, consistent with a primarily rumen-localised mode of action. These responses were associated with increases in weight gain (+7.96%; *p* = 0.002) and milk fat yield (+9.19%; *p* = 0.030). Meta-regression identified inclusion level and clay type as significant sources of variability (*p* < 0.05 for multiple outcomes). The findings indicate that clay supplementation supports methane abatement while improving rumen fermentation, digestibility, health, and productivity in ruminants.

## Simple Summary

This quantitative meta-analysis evaluated the effects of clay supplementation on rumen fermentation, digestibility, health, and productivity in ruminants. A total of 39 controlled studies were included in the dataset. The results showed that clay additives reduced methane and ammonia production while maintaining stability in overall ruminal functions. These responses are consistent with proposed mechanisms, although they were not directly evaluated in this meta-analysis. The digestibility of fibre and organic matter was moderately enhanced, accompanied by improvements in animal performance, such as weight gain and milk fat yield. These responses also varied depending on the type and dose of clay mineral. Collectively, clay minerals function as bioactive rumen modulators that enhance fermentation efficiency, improve nutrient utilisation, and support sustainable livestock production.

## 1. Introduction

Ruminants have long been observed to ingest soil and clay materials through natural geophagic behaviour [1,2,3]. Although the quantity consumed varies with animal species and environmental conditions, geophagic clay ingestion is generally episodic. It tends to occur during periods of mineral imbalance, toxin exposure, dietary stress, or gastrointestinal disturbance. In these situations, it serves as an adaptive behaviour for mineral acquisition, detoxification, and gastrointestinal regulation [1,3,4]. Such behaviour is widely interpreted as an adaptive response driven by physiological needs (e.g., mineral rebalancing, toxin adsorption, gut pH regulation), rather than a deliberate dietary choice by the animal [1,4]. Its persistence has led to the hypothesis that ingested clays and clay minerals may help stabilise rumen function by adsorbing inhibitory compounds, buffering physicochemical conditions, and supporting microbial activity [5,6]. These empirical observations stimulated scientific investigation into the use of clay minerals as functional feed additives, linking natural geophagic behaviour with modern nutritional research. Field observations of these responses have led to the deliberate provision of clay materials in grazing systems. Initially, clays were offered empirically to alleviate digestive disturbances and improve animal condition. Over time, this practice was formalised within animal nutrition, with clay minerals incorporated into formulated diets as functional feed additives designed to regulate rumen fermentation and enhance feed efficiency [7].

With advances in rumen microbiology and feed science, clays have gained increasing attention as modulators of ruminal processes. Materials such as bentonite, kaolin, montmorillonite, and zeolite have been extensively studied as naturally occurring clay-based feed additives in ruminant nutrition [6,7,8]. These materials differ considerably in mineralogical composition, cation-exchange capacity, swelling behaviour, surface area, and adsorption characteristics [1]. Because of these physicochemical differences, their interactions with ruminal substrates, metabolites, and microbial communities vary. As a result, different clay types can produce different effects on rumen fermentation, nutrient utilisation, and animal responses [6,9,10,11]. Through these interactions, clays appear to influence key rumen functions, including ammonia binding, microbial attachment, and ruminal physicochemical stability [1,6,11,12]. Consequently, dietary supplementation with clays has been associated with improved nitrogen utilisation, which may contribute to enhanced microbial protein synthesis. It can also increase fibre degradation through better microbial colonisation and enzymatic activity [6].

More recently, interest has extended to the role of clays in modulating hydrogen dynamics within the rumen [6,9,10,11]. Methane (CH_4_) formation is closely linked to hydrogen availability generated during fermentation. This process represents an energy loss to the host animal and contributes significantly to greenhouse gas emissions [13,14]. However, an effective methane mitigation strategy should reduce CH_4_ emissions without compromising rumen fermentation efficiency, nutrient utilisation, or animal productivity. This balance is essential for both environmental sustainability and efficient livestock production. By influencing microbial interactions and fermentation pathways, clays may redirect hydrogen towards alternative sinks such as propionate formation, while maintaining overall fermentation efficiency, including total volatile fatty acid production and ruminal pH [1,9,11]. However, methane mitigation represents only one component of their broader functional role of clays within the rumen ecosystem. Importantly, improving feed efficiency alongside enteric methane reduction offers dual economic and environmental benefits. Enhanced nutrient utilisation boosts animal productivity and reduces feed costs, while lower methane emissions decrease the environmental footprint. These combined benefits make clay supplementation a practical nutritional strategy for sustainable livestock production [1,11].

Despite these proposed mechanisms, experimental findings on the effects of clay supplementation remain inconsistent across animal species, experimental conditions (in vivo and in vitro), dietary composition, clay types, and supplementation levels [1,12,15,16]. This variability may stem from differences in forage-to-concentrate ratios, adaptation periods, animal physiological stages, rumen sampling methodologies, and basal diet composition. Some studies reported reductions in CH_4_ emissions alongside improvements in digestibility [9,10], whereas others showed minimal or variable effects on fermentation and animal performance [6,17]. This variability indicates that responses are context-dependent and influenced by both biological and physicochemical factors. Nevertheless, previous studies have reported maximum in vitro CH_4_ reductions of up to 23% with kaolin clay supplementation at approximately 5% dry matter intake. For in vivo trials, reductions reached ~13% using 1% natural illite. However, these responses varied according to clay type, inclusion level, and experimental conditions [9,10,12,15,16,17,18,19].

Previous reviews and meta-analyses have also examined CH_4_ mitigation strategies and rumen-modulating dietary interventions in ruminants, including feed inhibitors, tannin-rich diets, and broader nutritional approaches [8,20,21,22,23,24,25,26]. Collectively, these studies concluded that dietary interventions could reduce enteric methane emissions and, in some cases, improve rumen fermentation or animal performance. However, response magnitude varied considerably among additive types and experimental conditions. And these studies have primarily focused on specific additive classes or individual response domains. To our knowledge, no previous quantitative synthesis has simultaneously evaluated the effects of dietary clay supplementation on enteric methane emissions, rumen fermentation, nutrient digestibility, metabolic health indicators, and animal productivity. Furthermore, no prior study has identified key response moderators using meta-regression. Many published studies have been conducted with relatively small sample sizes and under diverse experimental conditions, including differences in animal species, clay type, inclusion level, study duration, and experimental design [15,27,28]. These factors contribute to inconsistent findings and may limit the statistical power of individual studies. 

Therefore, a quantitative meta-analysis is needed to estimate the overall effects of dietary clay supplementation and identifythe key moderators responsible for variation among studies. The primary objective of this study was to determine whether dietary clay supplementation reduces enteric methane emissions in ruminants. Secondary objectives were to evaluate its effects on rumen fermentation, nutrient digestibility, metabolic health indicators, and productivity. Finally, meta-regression analyses were conducted to evaluate how these responses vary with clay type, inclusion level, animal species, and experimental conditions (in vivo vs. in vitro) and other experimental design factors.

## 2. Materials and Methods

### 2.1. Literature Search and Study Selection

This systematic review and meta-analysis was conducted and reported in accordance with the Preferred Reporting Items for Systematic Reviews and Meta-Analyses (PRISMA 2020) guidelines (Checklist S1) [29]. The review protocol was not prospectively registered. A systematic literature search was conducted to identify peer-reviewed studies evaluating the effects of dietary clay and clay mineral supplementation in ruminants. Electronic searches were performed using the PubMed and Scopus databases, which provide broad coverage of peer-reviewed literature in animal science, veterinary science, nutrition, microbiology, and agricultural research, with the final search completed on 19 November 2025. These databases were selected because together they provide extensive coverage of the peer-reviewed literature relevant to the objectives of this review. To minimise the risk of missing eligible studies, supplementary searches were conducted using Google Scholar, and the first 10 pages of search results were screened. Reference lists of eligible publications were also examined. 

Only studies published in English were considered eligible for inclusion. The search was restricted to a ten-year publication window (2015–2025) to capture contemporary evidence on clay-based methane mitigation. Research evaluating the effects of dietary clay supplementation on enteric methane emissions is relatively recent. The earliest eligible study identified in our search was published by Tate, et al. [2], which proposed the potential of natural clay minerals as methane-mitigating feed additives based on the geophagic behaviour of grazing ruminants. Although eight studies published before 2015 were identified during the database search, these primarily investigated specific health or physiological outcomes and did not satisfy the predefined eligibility criteria for the quantitative synthesis. Moreover, no eligible studies addressing CH_4_ mitigation were identified before 2015. Therefore, inclusion of these earlier studies would not have altered the overall conclusions of the present review. The search strategy included combinations of keywords related to clay, clay minerals, kaolin, bentonite, montmorillonite, zeolite, ruminants, methane, rumen fermentation, digestibility, animal health, and productivity. A representative search string was: (“clay” OR kaolin OR kaolinite OR bentonite OR montmorillonite OR zeolite) AND (ruminant OR cattle OR cow* OR sheep OR goat*) AND (methane OR fermentation OR digestion OR productivity OR health). The complete database-specific search strategies and Boolean search strings are provided in Supplementary Figures S1 and S2.

The database search identified 51 records, comprising 20 records from PubMed and 31 records from Scopus (Figures S1 and S2). Following duplicate removal (*n* = 11), 40 records remained for title and abstract screening. Of these, 36 studies were subsequently retrieved and assessed for full-text eligibility. Among the full-text articles assessed, no studies were excluded solely because of the predefined 10-year publication window, as studies published before 2015 also failed to meet one or more predefined eligibility criteria. Studies were considered eligible when they: (i) evaluated natural clay minerals, including raw, purified, or commercially activated clay materials of natural origin, but excluding synthetic or chemically modified clay products; (ii) included an appropriate control group; (iii) reported quantitative outcomes related to rumen fermentation, methane emissions, digestibility, animal health, or productivity; and (iv) provided sufficient statistical information for effect size calculation. Studies were excluded when they: (i) used modified or engineered clay materials; (ii) evaluated clay supplementation in combination with other feed additives where the independent effect of clay could not be isolated; (iii) lacked an appropriate control group; or (iv) did not provide sufficient quantitative information for meta-analysis. Following title and abstract screening, four records were excluded because they involved modified clay materials or clay combined with other feed additives that did not meet the eligibility criteria.

**Figure 1:**
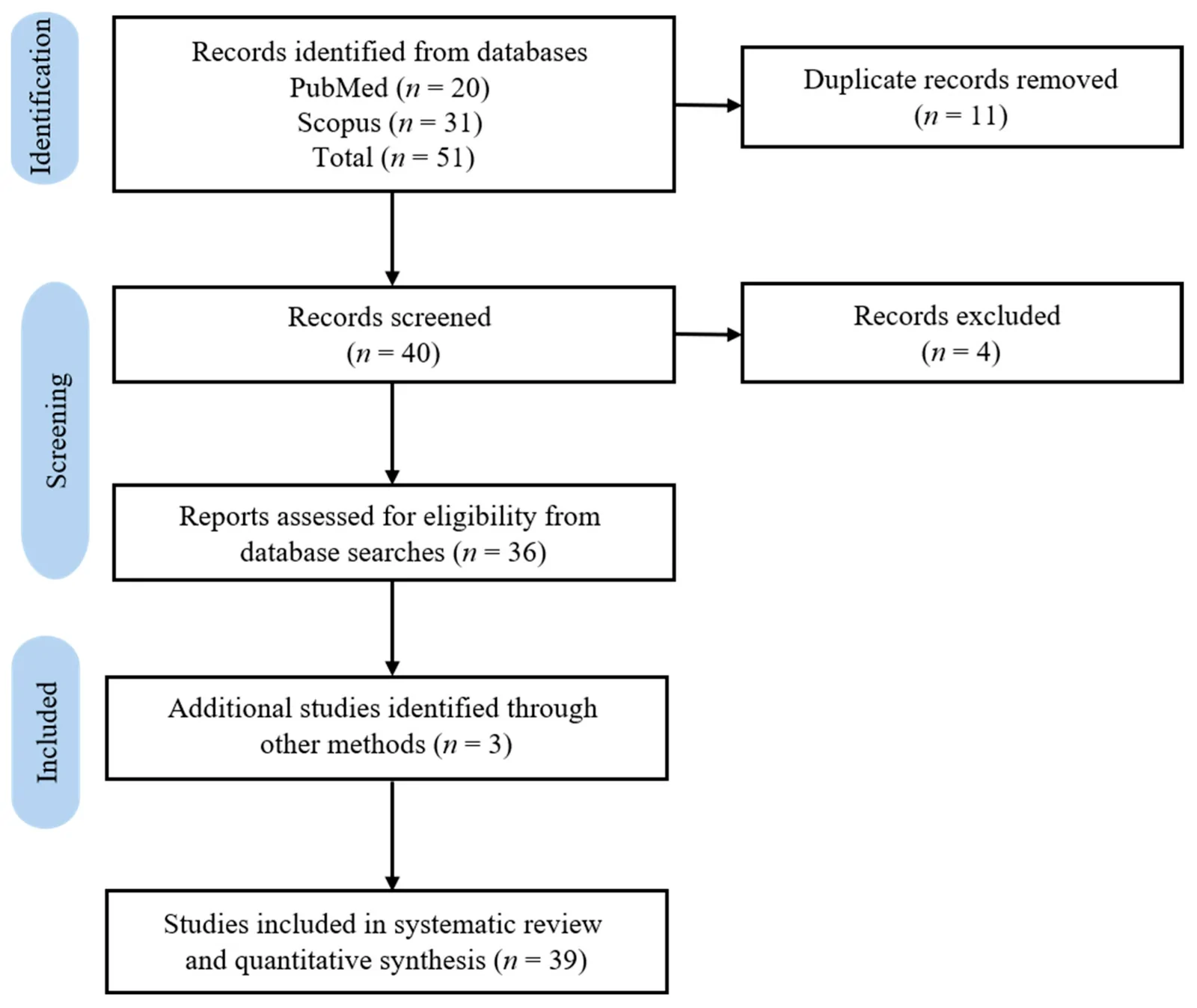
PRISMA 2020 flow diagram showing the study identification, screening, eligibility assessment, and inclusion process for the systematic review and quantitative meta-analysis.

To improve literature coverage and minimise the risk of omitting relevant studies not indexed in the primary databases, supplementary searches were conducted using Google Scholar and by screening the reference lists of eligible publications. This process identified three additional studies that met all inclusion criteria and were included in the quantitative synthesis. Consequently, a total of 39 studies were included in the final meta-analysis. The descriptive characteristics of all included studies are summarised in Table S1. Study selection and data extraction were independently performed manually by two authors. The included studies were also screened to identify multiple publications arising from the same experiment. No duplicate experimental datasets were identified, and all included studies represented independent experiments. Any discrepancies were resolved through discussion and verification against the original publications until consensus was reached. The complete study identification, screening, eligibility assessment, and inclusion process is illustrated in the PRISMA flow diagram (Figure 1).

### 2.2. Study Quality Assessment

A study quality assessment was conducted for all studies included in the quantitative synthesis. Study quality was evaluated using four methodological criteria: (i) presence of an appropriate control group, (ii) random allocation of animals or experimental units to treatments, (iii) adequacy of replication, and (iv) reporting of variance measures (SD or SEM). Because this review included both in vivo and in vitro studies with heterogeneous experimental designs, a simplified quality assessment based on key methodological criteria was considered more appropriate than study design-specific risk-of-bias tools.

Each criterion was classified as “low risk” when it was adequately reported in the original study. Where methodological details were unclear or insufficiently reported, the criterion was classified as having “some concerns”. No studies were classified as “high risk” based on the predefined assessment criteria. The assessment was independently performed by two authors, and any discrepancies were resolved through discussion and consensus. A summary of the study quality assessment is presented in Table S2. Because study protocols or pre-registered analysis plans were generally unavailable, selective outcome reporting could not be reliably assessed and was therefore excluded from the study quality evaluation.

### 2.3. Certainty of Evidence Assessment

The certainty of evidence was evaluated using the Grading of Recommendations, Assessment, Development, and Evaluations (GRADE) approach, which has been adapted for evidence synthesis in animal nutrition to provide a transparent and structured framework for evaluating the certainty of evidence across outcomes. Overall certainty ratings were assigned based on the combined assessment of five key domains: risk-of-bias, inconsistency, indirectness, imprecision, and publication bias. “Risk-of-bias” was assessed using the predefined study quality criteria (Table S2). “Inconsistency” was informed by evaluating statistical study heterogeneity via I^2^ and τ^2^ statistics. “Indirectness” was determined by the relevance of included studies to the review objectives, while “imprecision” was judged by the number of studies and confidence intervals. Finally, “publication bias” was evaluated using Egger’s regression tests and trim-and-fill analyses.

### 2.4. Data Extraction and Classification

From each eligible study, the following experimental conditions were recorded: ruminant species, production type, clay type, inclusion level (g/kg DM), duration, and study type (in vivo or in vitro). Unless otherwise specified, the term “clay” is used throughout this manuscript as a collective descriptor for clay minerals and zeolites included in the reviewed literature. Specific mineral types are identified separately where relevant. Quantitative outcome variables extracted for meta-analysis included methane production, total gas production, ruminal pH, total volatile fatty acids, acetate, propionate, butyrate, and ammonia-N. Among digestibility indices, dry matter, organic matter, neutral detergent fibre, acid detergent fibre, and crude protein digestibility were recorded. Other variables such as blood biochemical parameters (e.g., blood urea nitrogen, glucose, and liver enzymes), and productivity measures, including milk yield, milk composition, feed intake, and weight gain, were also extracted. The means, along with their corresponding measures of variance (SD or SEM) and sample sizes, were extracted for effect size calculation. Quantitative outcomes were extracted as means ± standard error of the mean (SEM) and sample sizes for control/treatment groups; standard deviation (SD) was derived from SEM as SD = SEM × √*n*, where *n* is the sample size [30,31]. All included studies reported variance as either SD or SEM; therefore, no studies were excluded because of missing variance data. Where outcome variables were reported using different units across studies, values were converted to common standard units before analysis to ensure consistency and comparability across studies. Outcomes were classified into four domains: rumen fermentation, digestibility, blood/metabolic health indicators, and productivity.

**Figure 2:**
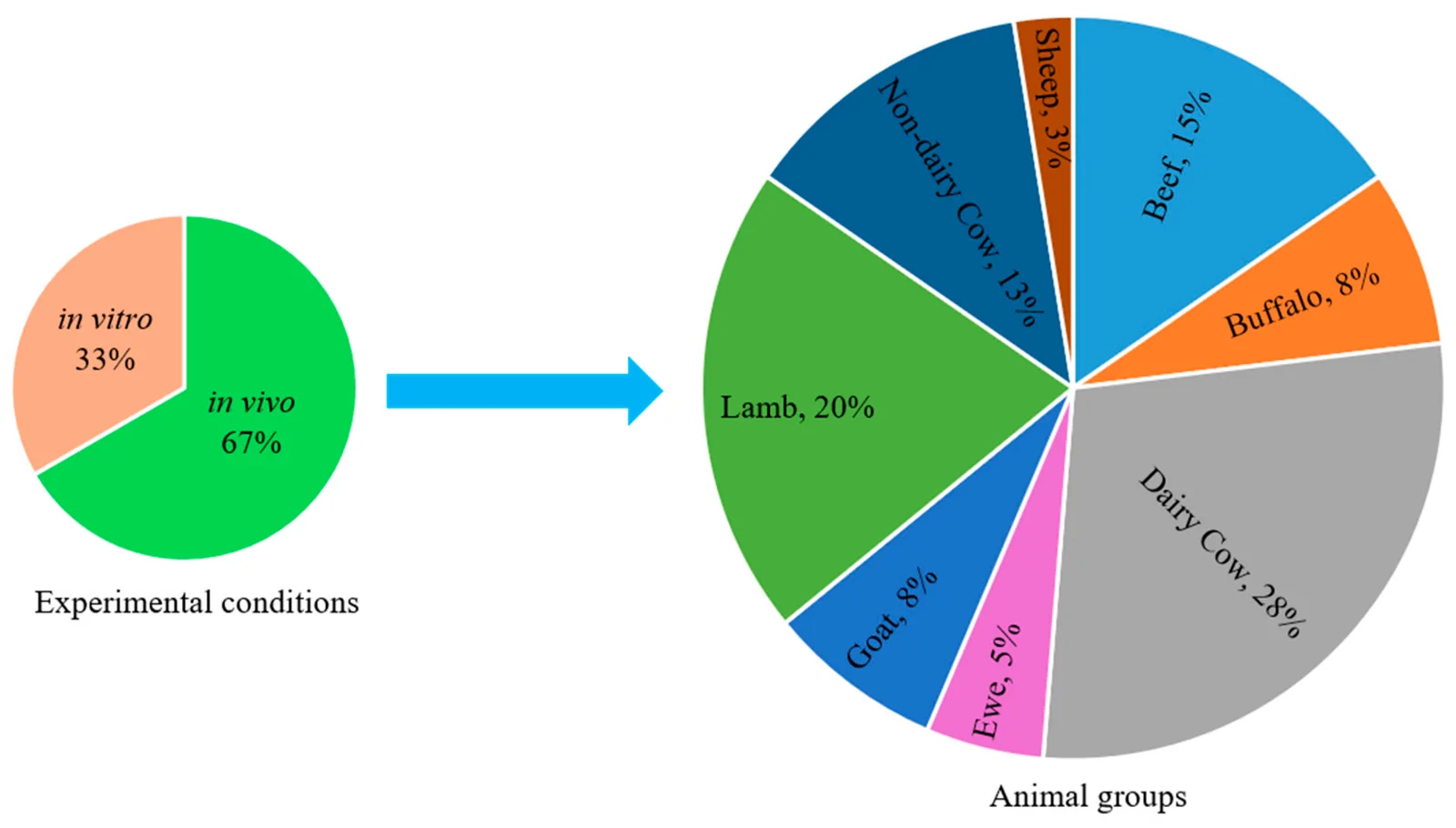
Distribution of included studies by experimental system (in vivo vs. in vitro). Data are presented as reported in the original studies without modification.

The study distribution by experimental system is shown in Figure 2. The composition of clays and clay minerals (Table S3) and the characteristics of basal diets (Table S4) summarise the variability across studies. Clay supplementation ranged from 0.2 to 62.5 g/kg dry matter across different ruminant species. Because clay minerals were obtained from different natural deposits and geographic regions, their mineralogical and chemical compositions varied substantially among studies. The compositions summarised in Table S3 therefore represent only the clay materials evaluated in the included studies and should not be considered representative of all naturally occurring clay deposits worldwide. This variability may contribute to differences in biological responses observed across studies. However, mineral composition was not included as a moderator because detailed mineralogical characterisation was not reported consistently across the included studies. Similarly, clay supplementation levels ranged from 0.2 to 62.5 g/kg dry matter, providing a broad range for evaluating dose-dependent responses. The influence of supplementation level on response variability was subsequently examined through meta-regression analyses.

Analytical methods used to quantify methane emissions, fermentation parameters, digestibility, blood metabolites, and productivity traits were extracted from each study. Techniques included gas chromatography or semi-automatic gas production systems for methane and volatile fatty acids, spectrophotometric assays for ammonia nitrogen and blood indices, total collection or marker-based methods for digestibility, and automated milk analysers for production traits. A summary of analytical approaches across domains is provided in Table S5. Analytical methods were extracted to document methodological diversity across studies but were not included as moderators because the available data were insufficient for robust subgroup or meta-regression analyses.

Species categories were harmonised prior to modelling to ensure sufficient statistical power and biological interpretability. Although study characteristics are presented in detail (e.g., dairy cow, non-dairy cow, beef cattle, lamb, sheep, ewe) within descriptive tables (Table S1), related production types were grouped during meta-regression analysis to avoid sparse-category bias and unstable variance estimates. Specifically, beef cattle and non-dairy cattle were combined under the category “Beef,” while sheep, lamb, and ewe were consolidated under “Sheep.” Dairy cattle, buffalo and goats were retained as separate categories due to their distinct species, metabolic demands, and production characteristics. This harmonisation approach aligns with established recommendations for moderator analysis in meta-regression, where biologically comparable subgroups are combined to improve model stability while preserving interpretive relevance [32,33]. However, pooling beef cattle with non-dairy cattle may retain some biological heterogeneity because differences in growth stage and physiological status could not be fully accounted for using the available data.

### 2.5. Effect Size Calculation

Effect sizes were calculated following the previous reports [34,35]. The natural logarithm of the response ratio (lnRR) was used, as follows:

$$lnRR=\ln\left(\frac{{Mean}_{treatment}}{{Mean}_{control}}\right)$$

here, “Outcomes” were included when valid Control–Treatment comparisons were available with sufficient quantitative data for the calculation of effect size, regardless of the direction (increase or decrease) of the response. Here “Outcome” refers to the measured result for each variable studied. The log response ratio (lnRR) was selected because it expresses “Treatment” effects as proportional changes relative to the “Control”, allowing direct biological interpretation across obtained results and the ruminant species studied. This approach is widely used in ecological, agricultural, and animal nutrition meta-analyses where “Outcomes” are measured on different scales [35].

Sampling variance of lnRR (v) was also calculated using the following equation:

$$v=\left(\frac{{SD}_{t^{2}}}{n_{t}\times {Mean}_{t^{2\;}}}\right)+\;\left(\frac{{SD}_{c^{2}}}{n_{c}\times {Mean}_{c^{2\;}}}\right)$$

where ${SD}_{t}$ and ${SD}_{t}$ represent standard deviations, and $n_{t}$ and $n_{c}$ represent sample sizes for Treatment and Control.

Percent change relative to Control:

$$Percent\;change=\left(e^{lnRR}-1\right)\times 100$$

### 2.6. Meta-Analytical Models

Random-effects meta-analytic models were used to estimate the pooled effects of clay supplementation across studies. Multilevel random-effects structures were specified to account for non-independence among effect sizes arising from the same study, with study identity and comparison type (in vivo vs. in vitro) included as random effects. Where a study included multiple treatment groups sharing a common control group, the dependency among effect sizes was accounted for within the multilevel random-effects model. Restricted maximum likelihood (REML) estimation was applied to obtain pooled effects and 95% confidence intervals (CI) [36]. Separate models were fitted for each outcome domain in accordance with current methodological recommendations for hierarchical meta-analysis [37]. To explore the influence of experimental system, predefined subgroup analyses were conducted separately for in vivo and in vitro studies for the rumen fermentation and digestibility outcomes. Separate random-effects models were fitted for each subgroup to evaluate whether the magnitude and direction of responses differed between experimental conditions. Health and productivity outcomes were not subjected to subgroup analyses because all eligible studies were conducted in vivo. In addition, study type (in vivo vs. in vitro) was included as both a random-effect grouping factor and a moderator in the meta-regression models to evaluate its contribution to between-study heterogeneity. Moderators were evaluated individually in separate meta-regression models; therefore, multicollinearity among moderators was not assessed.

Further, to investigate potential sources of between-study heterogeneity, mixed-effects meta-regression analyses were conducted using biologically relevant moderators, including clay type, ruminant species, clay inclusion level, and experimental duration. In these models, clay inclusion level was treated as a categorical moderator and classified as low (<10 g/kg DM), medium (10–20 g/kg DM), and high (>20 g/kg DM). Inclusion of species as a moderator was considered particularly important to account for biological differences among ruminant species and to minimise potential bias associated with pooling data across different species. Moderator effects were evaluated using mixed-effects models fitted with the metafor package (v4.8.0) for R software, and statistical significance was assessed using Wald-type tests. Where sufficient observations were available, both univariable and adjusted multivariable models were examined.

### 2.7. Statistical Software

All analyses were conducted in R 4.5.2 using packages metafor (v4.8.0, multilevel meta-analysis), dplyr (v1.2.0, data conversion), and ggplot2 (v4.0.2, visualisations). Effect sizes, moderator analyses, and forest plots were generated within a unified workflow to ensure consistency across outcome domains. These packages provide estimates of heterogeneity, robust visualisation tools, and functions to explore publication bias [38].

### 2.8. Publication Bias and Sensitivity Assessment

Publication bias and sensitivity analyses were performed after fitting the primary meta-analytic models to assess the robustness of statistically significant pooled outcomes. Small-study effects were evaluated for all statistically significant outcomes using funnel plot inspection, Egger’s regression test (*p* < 0.05 threshold) [39], and Duval & Tweedie’s trim-and-fill analysis [40]. Egger’s regression test was performed only for outcomes with ≥10 effect sizes to ensure reliable assessment of small-study effects. In these cases contemporary recommendations for bias-adjusted inference under heterogeneity were followed [41]. For example, funnel plots were constructed from multilevel random-effects models employing log-response ratios (yi) and corresponding sampling variances (vi). Egger’s test regressed standardised effect sizes against study precision (1/√vi) to quantify asymmetry [39]. Statistically significant asymmetry triggered trim-and-fill procedures to estimate potentially missing studies and recalculate bias-adjusted pooled effects [40,42]. These assessments were conducted independently across fermentation, digestibility, health, and productivity outcome domains. Sensitivity analyses comprised leave-one-study-out cross-validation, influence diagnostics (Cook’s distance > 4/(k − 1), DFFITS > 2√(k/*n*)), and sequential exclusion of the highest-weighted studies [32]. All analyses were conducted in R 4.5.2 using the metafor package (v4.8.0).

## 3. Results

### 3.1. Effects of Clay Supplementation on Enteric Fermentation

Clay supplementation significantly reduced CH_4_ production by 20.45% (*p* < 0.001) across studies with supplementation levels ranging from 0.2 to 62.5 g/kg DM, while total gas production remained unchanged (*p* = 0.27) (Table 1). Ruminal pH was unaffected (*p* = 0.75), indicating no disruption to rumen acid–base balance. Total volatile fatty acids (VFA) concentrations were not significantly altered overall; however, propionate increased modestly (+4.35%; *p* = 0.046), and NH_3_-N concentrations decreased (−11.51%; *p* = 0.033). Acetate, butyrate, and minor VFA fractions showed no significant changes (Table 1). Subgroup analyses according to experimental condition (Table S6) showed that clay supplementation reduced CH_4_ production by 21.94% (*p* < 0.001) in in vitro studies, whereas the reduction observed in in vivo studies (−16.25%) was not statistically significant (*p* = 0.358). Total VFA concentrations increased by 6.32% (*p* = 0.012) in in vivo studies but remained unchanged in vitro, while propionate increased only in vitro (+6.49%, *p* < 0.001). In contrast, butyrate increased only in in vivo studies (+7.08%, *p* = 0.041). NH_3_-N concentrations decreased similarly in both in vivo (−15.01%, *p* = 0.039) and in vitro (−15.38%, *p* = 0.050) studies. Protozoal abundance increased by 23.35% (*p* < 0.001) in in vitro studies but was unaffected in vivo. The remaining fermentation parameters did not differ significantly between clay-supplemented and control groups within either experimental condition. Meta-regression analyses, which adjusted for inclusion level, species, and clay types as covariates, revealed that several of these moderators significantly influenced rumen fermentation responses beyond the overall pooled effects (Table S7).

This analysis also identified clay inclusion level, ruminant species, and clay mineralogy as important sources of between-study heterogeneity (Table S7). Clay inclusion level showed consistent dose-dependent effects across multiple outcomes, with reductions observed in acetate (−0.21% per 1 g/kg DM, *p* < 0.001), CH_4_ (−1.51% per 1 g/kg DM, *p* < 0.001), protozoa (−1.58% per 1 g/kg DM, *p* < 0.001), and total gas (−0.64% per 1 g/kg DM, *p* < 0.001), while increases were observed in NH_3_-N (0.65% per 1 g/kg DM, *p* = 0.001) and butyrate (0.18% per 1 g/kg DM, *p* = 0.014). Species-specific responses were observed particularly for acetate, which decreased in both cattle (−15.45%, *p* = 0.045) and sheep (−16.92%, *p* = 0.033).

Clay mineralogy also significantly influenced several fermentation parameters. For example, saponite increased CH_4_ production (+54.68%, *p* = 0.003) but reduced butyrate (−37.70%, *p* = 0.001) and total VFA (−27.56%, *p* < 0.001). Illite, kaolin, and montmorillonite decreased pH (−11.05%, −10.49%, and −5.95%, respectively; *p* ≤ 0.05), whereas sepiolite and zeolite reduced valerate concentrations (−23.10% and −21.86%, respectively; *p* < 0.001). Zeolite-based materials were additionally associated with increased protozoal abundance (+29.23% and +82.74%, respectively; *p* < 0.001). In contrast, propionate and isobutyrate were generally less responsive to differences in clay mineralogy. Overall, clay supplementation reduced enteric methane production and ammonia-nitrogen concentrations while maintaining overall rumen fermentation functions.

### 3.2. Influences of Clay Supplementation on Rumen Digestibility

**Figure 3:**
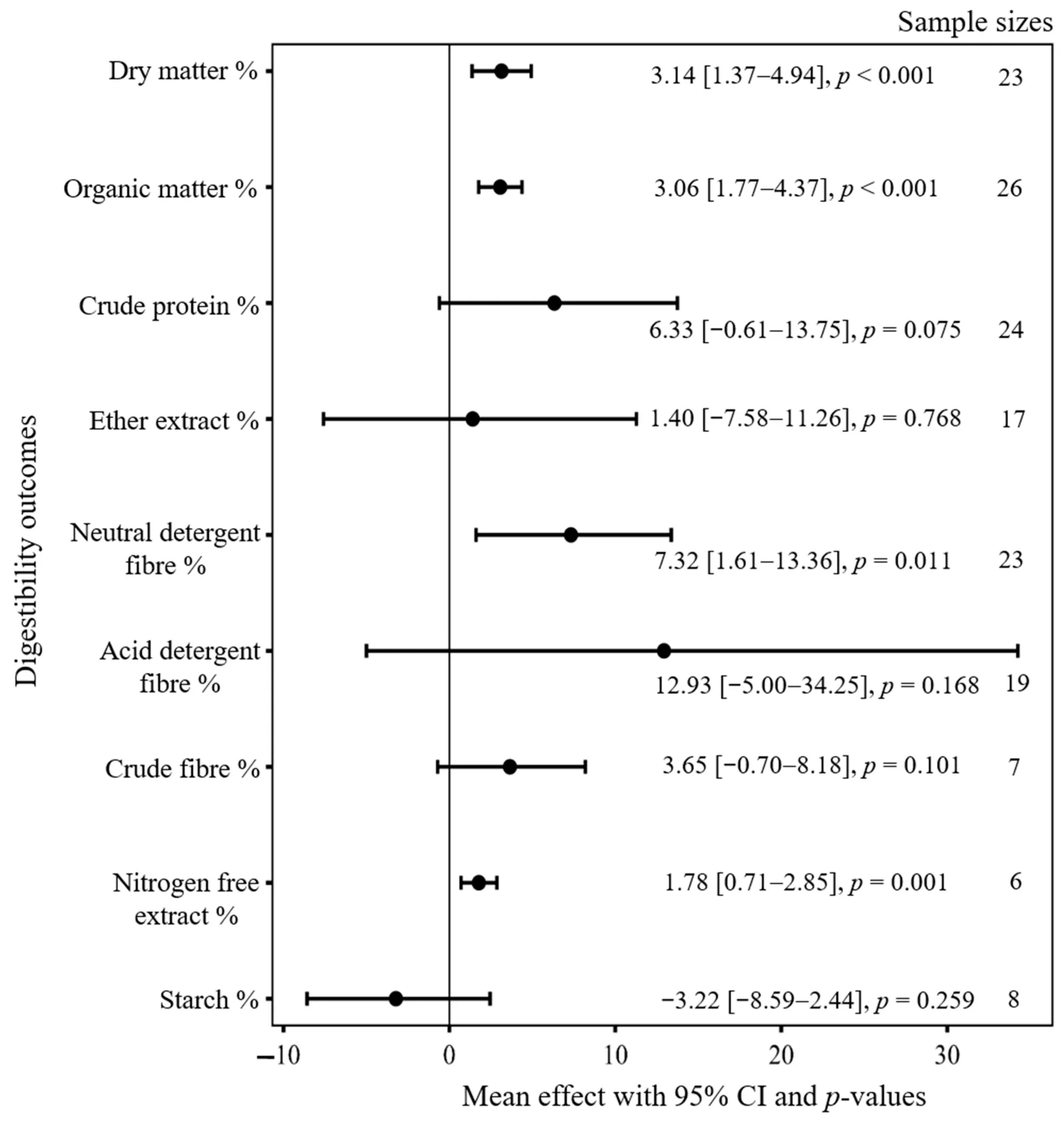
Relative effects of clay supplementation on digestibility outcomes across studies, presented as relative effects (%) with corresponding upper and lower 95% confidence intervals and *p*-values compared with control. The larger error bars reflect the true between-study variability (heterogeneity) rather than a measurement error within a single experiment. Here, effect sizes represent number of studies.

Several digestibility parameters showed significant positive responses, particularly for dry matter, organic matter, and fibre digestibility (Figure 3). For example, dry matter digestibility increased by +3.14% (*p* < 0.001), organic matter digestibility by +3.06% (*p* < 0.001), neutral detergent fibre digestibility by +7.32% (*p* = 0.011), and nitrogen-free extract by +1.77% (*p* = 0.001). Crude protein, ether extract, acid detergent fibre, crude fibre, and starch digestibility were not significantly affected by clay supplementation (*p* = 0.075, 0.769, 0.168, 0.101, and 0.259, respectively) (Figure 3).

Subgroup analyses according to experimental condition (Table S8) showed that clay supplementation significantly improved digestibility of DM (+3.32%, *p* < 0.001), OM (+3.17%, *p* < 0.001), CP (+6.07%, *p* = 0.021), NDF (+8.99%, *p* = 0.002), and ADF (+18.41%, *p* = 0.041) in in vivo studies. No significant effects were observed for EE, CF, NFE, or starch digestibility. In in vitro studies, clay supplementation did not significantly affect OM, NDF, or ADF digestibility, although the positive response for ADF approached statistical significance (*p* = 0.054). Meta-regression analysis demonstrated that clay type and inclusion level were the primary drivers of variation in digestibility outcomes (Table S9). For organic matter digestibility, montmorillonite was positively associated with improved digestibility (β = 0.006, *p* = 0.005), whereas zeolite was associated with reduced digestibility relative to the reference category (β = −0.032, *p* = 0.001). A similar pattern was observed for crude protein digestibility when these two minerals were included (β = 0.032, *p* < 0.001 for montmorillonite; β = −0.038, *p* = 0.013 for zeolite); either case, a significant negative dose–response relationship was also observed (β = −0.005 per unit, *p* < 0.001). Ether extract digestibility was positively associated with montmorillonite (β = 0.026, *p* < 0.001), whereas zeolite supplementation was associated with reduced ether extract digestibility responses (β = −0.091 and −0.034, *p* = 0.027). In this case also, the supplementation level exerted a significant negative effect (β = −0.007, *p* < 0.001).

Neutral detergent fibre digestibility was positively influenced by montmorillonite (β = 0.042, *p* = 0.035) but declined significantly with increasing inclusion level (β = −0.005, *p* < 0.001). This effect was further exacerbated for acid detergent fibre digestibility, where it showed a strong negative dose effect (β = −0.046, *p* < 0.001). Dry matter digestibility was found to be increased with the inclusion of montmorillonite (β = 0.011, *p* < 0.001) and decreased with zeolite (β = −0.036, *p* < 0.001). In both cases, data regarding inclusion level were unavailable. Crude fibre digestibility was also reduced by the inclusion of zeolite (β = −0.043, *p* = 0.036), and there was no information recorded for its dosage. However, it was found that this digestibility was negatively associated with increasing clay inclusion level (β = −0.098, *p* = 0.002).

Further, zeolite and its incremental dosage were found to be negatively associated with nitrogen-free extract digestibility outcome (β = −0.019, *p* = 0.024, and β = −0.071, *p* = 0.001, respectively). In contrast, starch digestibility exhibited a strong positive dose–response (β = 0.007, *p* < 0.001), suggesting differential substrate-specific responses to clay inclusion. Species effects were generally limited across digestibility outcomes. However, crude fibre digestibility was positively associated with cattle (β = 0.99, *p* = 0.002) and sheep (β = 1.89, *p* = 0.003), whereas nitrogen-free extract digestibility showed positive associations with cattle (β = 0.73, *p* = 0.001) and goats (β = 0.09, *p* = 0.012) (Table S9). No significant species effects were detected for organic matter, crude protein, ether extract, neutral detergent fibre, acid detergent fibre, dry matter, or starch digestibility. These findings suggest that clay supplementation generally enhanced nutrient digestibility, particularly that involving fibre and dry matter.

### 3.3. Impacts of Clays on Metabolic Health Indicators Across Studies

Among blood metabolites, only blood urea nitrogen (BUN) showed a significant reduction (−5.64%; *p* = 0.002). Albumin, total protein, glucose, alanine aminotransferase (ALT), aspartate aminotransferase (AST), creatinine, triglycerides, and electrolyte concentrations were not significantly altered (*p* = 0.510, 0.418, 0.344, 0.647, 0.961, 0.054, and 0.257, respectively; sodium, potassium, and chloride: *p* = 0.681, 0.896, and 0.377, respectively) (Table 2).

Here, the adjusted meta-regression analyses (Table S10) indicated that responses were primarily driven by clay type, species, and inclusion level. Montmorillonite-based materials markedly reduced bilirubin (−97.77%; *p* < 0.001) and total cholesterol (−18.99%; *p* = 0.002), while nano-zeolite also lowered total cholesterol (−21.96%; *p* < 0.001) and altered glucose responses. It is worth noting that the meta-analysis was unable to define the exact size of zeolite; hence, the term “nano-zeolite” material is retained as reported in the original studies. Typically, “nano-zeolite” refers to particles smaller than 100 nm in at least one dimension. Globulin decreased significantly in goats (−15.68%; *p* = 0.002), and creatinine was reduced in sheep (−13.32%; *p* = 0.030). Dose-dependent effects were evident for several traits, including reductions in bilirubin (−53.62% per unit; *p* < 0.001) and lactose content (−0.48% per unit; *p* < 0.001), alongside increases in milk fat (+1.89% per unit; *p* < 0.001) and protein content (+1.46% per unit; *p* < 0.001). The health-related outcomes caused by clay supplement indicate limited effects on most blood metabolites, although reductions in blood urea nitrogen were observed.

### 3.4. Functional Role of Clays and Clay Minerals on Ruminants’ Productivity

**Figure 4:**
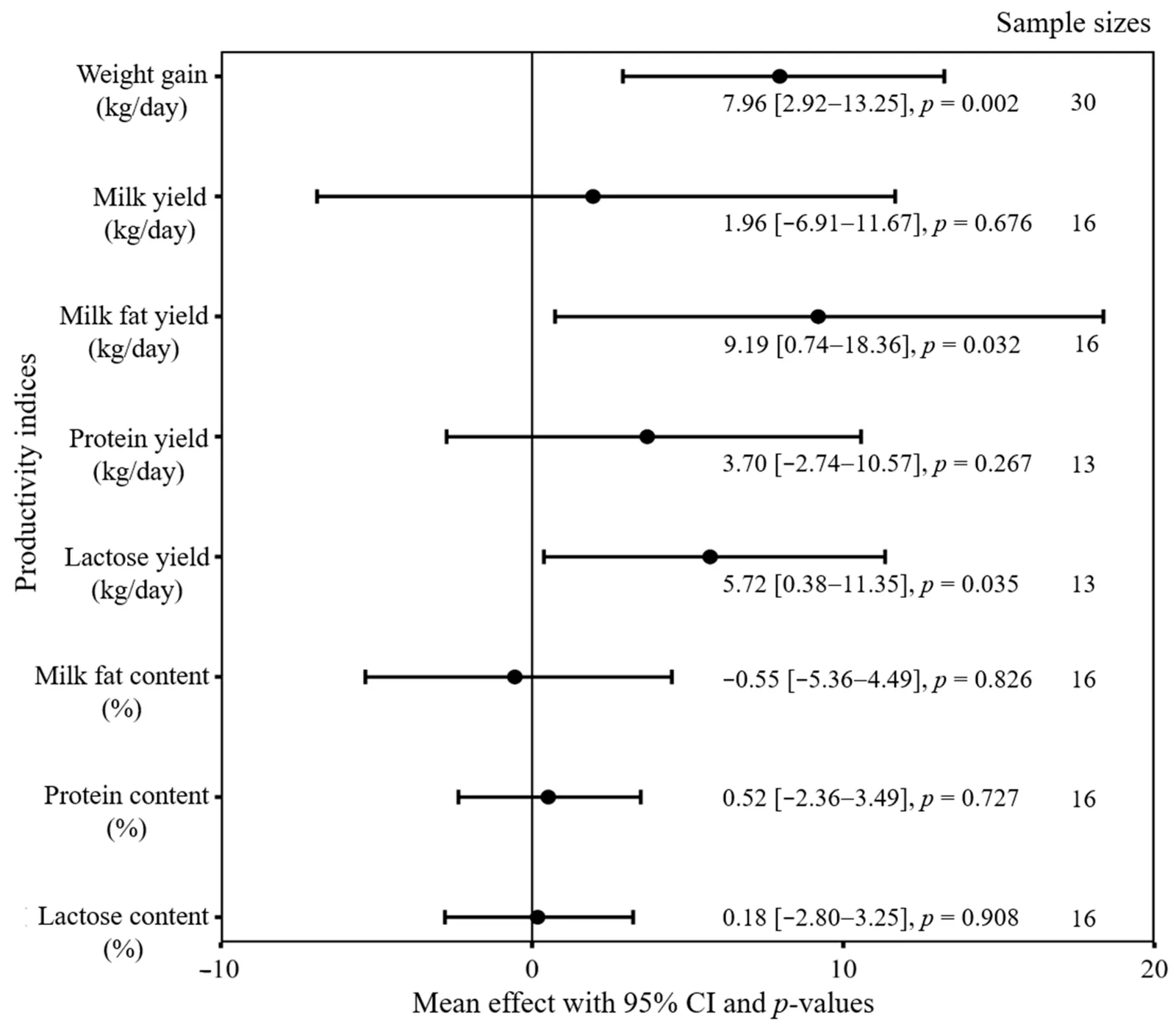
Relative effects of clay supplementation on productivity outcomes across studies, presented as relative effect (%) with corresponding upper and lower 95% confidence intervals and *p*-values relative to the control. The larger error bars reflect the true between-study variability (heterogeneity) rather than measurement error within a single experiment. Effect sizes correspond to the number of studies included in each analysis.

Clay supplementation significantly increased weight gain by +7.96% (*p* = 0.002; *n* = 30). Among milk production traits, fat yield increased by +9.19% (*p* = 0.032; *n* = 16) and lactose yield increased by +5.72% (*p* = 0.035; *n* = 13) (Figure 4).

The adjusted meta-regression models showed that several moderators significantly influenced productivity responses (Table S11). Clay inclusion level exhibited a strong negative association with weight gain (estimate = −0.004; *p* < 0.001), indicating diminishing marginal gains at higher inclusion levels. Milk yield was significantly influenced by supplement type, with zeolite supplementation associated with a lower milk yield response relative to other additives (estimate = −0.112; *p* < 0.001). Fat yield responses varied according to clay type and supplementation duration. In this case, negative effects were observed for certain clay categories (*p* < 0.001), whereas duration showed a positive association with fat yield (estimate = 0.002; *p* = 0.022). Protein yield was also significantly modified by mineral types, with montmorillonite associated with an increased response (estimate = 0.102; *p* = 0.004) and zeolite associated with a reduced response (estimate = −0.166; *p* < 0.001). Lactose yield was similarly influenced by clay type, particularly zeolite (estimate = −0.145; *p* < 0.001).

Milk composition also showed a moderator effect. Fat content in milk increased in studies using nano-zeolite materials (estimate = 0.315; *p* < 0.001) and with increasing supplementation level (estimate = 0.019; *p* < 0.001). In contrast, studies using “zeolite” reported in standard particle size (>100 nm) showed a reduction in fat content (estimate = −0.178; *p* < 0.001). Montmorillonite and nano-zeolite (<100 nm) showed positive associations for protein content, while natural zeolite (bulk size, i.e., not nano-zeolite) showed a negative association (*p* < 0.001). Lactose content was significantly affected by species, supplement type, inclusion level, and study duration. Higher lactose content was associated with goats (*p* = 0.002) and longer study duration (estimate = 0.0005; *p* = 0.035). In contrast, lower lactose content was associated with montmorillonite and nano-zeolite (<100 nm) (*p* < 0.001) and with increasing inclusion level (estimate = −0.005; *p* < 0.001). Taken together, the productivity responses suggest that clay supplementation may contribute to improvements in animal performance, particularly weight gain and selected production traits.

### 3.5. Publication Bias and Sensitivity Analyses

Funnel plot inspection, Egger’s regression tests, and trim-and-fill analyses indicated evidence of publication bias for a subset of outcomes, whereas several key variables, including CH_4_ and BUN, showed little evidence of asymmetry (Table 3). For example, CH_4_ production (z = 1.83, *p* = 0.067, k = 85) did not require trim-and-fill imputation. Significant asymmetry (*p* < 0.05) occurred in 7 out of 11 outcomes, notably fermentation intermediates (NH_3_-N z = 4.32; propionate z = 4.35) and all productivity traits (weight gain *p* = 0.028; fat yield *p* = 0.008; lactose yield *p* < 0.001). Neutral detergent fibre digestibility required substantial adjustment (+40 imputed studies), while blood urea nitrogen showed symmetry (*p* = 0.256). Trim-and-fill analysis imputed 0–39 studies across all outcome domains, with productivity outcomes exhibiting moderate adjustments (Table 3).

Conversely, leave-one-out cross-validation confirmed stability across all 11 outcomes (Table 4). Methane production showed minimal variation (−23.67%; range: −25.65 to −20.69%) with no influential studies. Digestibility parameters showed relatively narrow response ranges (dry matter digestibility: 3.28–3.81%; organic matter digestibility: 2.97–3.32%; NDF digestibility: 7.37–9.48%), all remaining below predefined influence thresholds. Although the NDF response exhibited a numerically wider absolute range (2.11 percentage points) compared with weight gain (1.21 percentage points), variability is more appropriately evaluated using heterogeneity statistics rather than absolute range alone. In the primary meta-analytic model (Table 2), NDF digestibility was not statistically significant (*p* = 0.653), whereas weight gain demonstrated a significant positive effect (*p* = 0.028).

No outcome changed direction or lost significance following individual study removal, confirming the robustness of all pooled estimates to single-study effects. Pooled effects changed by less than 3% on average (maximum 4.58%), meeting stringent criteria for meta-analytic stability.

Although publication bias was detected for some outcomes, the pooled estimates remained stable in the sensitivity analyses. No influential studies were identified.

### 3.6. Certainty of Evidence Across Studies

The certainty-of-evidence assessment indicated low to moderate confidence across the major outcome domains evaluated in this review (Table S12). Moderate certainty was assigned to rumen fermentation and methane mitigation outcomes, whereas nutrient digestibility, animal health, and productive performance outcomes were assigned low certainty. The primary factors reducing certainty were “between-study heterogeneity” and evidence of publication bias for selected outcomes. These findings suggest that the overall body of evidence should be interpreted with caution despite the generally robust pooled estimates.

## 4. Discussion

Rumen fermentation responses to clay supplementation reflect changes in rumen fermentation efficiency rather than suppression of overall microbial activity. These responses are consistent with the proposed hydrogen and nitrogen metabolism pathway, favouring reduced methanogenesis and improved nutrient capture while maintaining overall fermentation stability [1,10]. The outcomes from the subgroup analyses suggest that the magnitude of the fermentation response may differ between experimental conditions. These findings highlight the importance of validating in vitro observations under in vivo conditions. Species was also evaluated as a potential influencing factor across most outcome domains. However, significant species effects were detected only for selected variables, including acetate concentration, crude fibre digestibility, nitrogen-free extract, creatinine, globulin, and lactose content (Tables S6–S11). In contrast, methane- and nitrogen-related responses were not significantly influenced by species, suggesting that these responses were generally consistent across the included ruminant species and appeared to be more strongly associated with mineral (e.g., clay mineral and zeolite) supplementation. However, the limited number of studies available for some species may have reduced the statistical power to detect biologically meaningful species-specific differences.

Such targeted responses are particularly evident in kaolin-based in vitro experiments, where CH_4_ reduction occurs without significant alterations in ruminal pH, redox conditions, or other core fermentation parameters. This is consistent with proposed hydrogen redirection mechanisms and surface adsorption of dissolved hydrogen or derived methane, rather than broad microbial inhibition [9,11]. Supplementation level was also found to be the strongest predictor of CH_4_ mitigation. This is consistent with the observed 20.45% reduction in CH_4_ production (Table 1), confirming a substantial fermentation shift under clay supplementation. Further, meta-regression results (Table S6) showed a significant negative association between inclusion level and CH_4_ (−1.51% per 1 g/kg DM increase, *p* < 0.001), supporting its role as a key driver of response variability. This dose-dependent pattern is supported by individual studies reporting greater methane reduction at higher inclusion levels of clay-based additives [1,43]. To place the observed methane reduction in the context of other established dietary mitigation strategies, Table 5 summarises the approximate methane reduction reported for major feed additive categories based on published meta-analyses and systematic reviews.

As shown in Table 5, the methane reduction achieved with clay supplementation (20.45%) is comparable with or greater than that reported for several natural feed additives, such as tannins, while remaining lower than highly effective inhibitors such as 3-nitrooxypropanol (3-NOP) and *Asparagopsis* spp. These findings suggest that hydrogen utilisation pathways are sensitive to clay inclusion level, highlighting supplementation level as an important determinant of methane mitigation responses [43,44]. However, this effect is not universal across studies. Studies have reported minimal or inconsistent CH_4_ responses despite increasing supplementation levels. In this case, diet composition, rumen conditions, and/or availability of methane-generating substrate may be a dominant factor(s) over the inclusion level under certain conditions [21,45]. This highlights that supplementation level is an important but context-dependent driver of CH_4_ mitigation.

Digestibility responses further highlight the functional role of clays in the rumen. Improvements in fibre and organic matter digestibility indicate enhanced microbial colonisation and substrate degradation [46]. However, the effects of clay supplementation on rumen metabolism are likely multifactorial and may vary according to mineral type, inclusion level, and experimental conditions [1,10]. The subgroup analyses indicate that digestibility responses were more evident in vivo. Further in vitro studies are needed to strengthen the available evidence. Potential mechanisms include modulation of microbial activity, altered nutrient availability, improved rumen physicochemical conditions, and selective adsorption of metabolites or inhibitory compounds [10,46]. These responses are consistent with previous studies demonstrating improved fibre utilisation and fermentation efficiency in clay-supplemented diets, particularly under high-concentrate feeding conditions [6,15,16]. Here, the stabilisation of rumen pH through ion exchange and adsorption mechanisms likely creates favourable conditions for fibrolytic microorganisms, supporting enhanced fibre degradation without suppressing total fermentation activity. For instance, Al Adawi, et al. [47], Gallo, et al. [48], and Jiang, et al. [49] reported improved digestibility and nutrient utilisation following supplementation with modified montmorillonite, bentonite, and other clay-based additives under different experimental conditions. Although these individual studies differed in clay type, animal species, and production system, their findings are broadly consistent with the overall trends observed in the present meta-analysis [7,28]. However, improvements in digestibility do not necessarily translate into proportional gains in animal productivity, as productive performance is also influenced by factors such as physiological stage, nutrient partitioning, diet composition, and management conditions [50].

Despite these positive responses, variability across studies remains significant, as reflected by the heterogeneity statistics reported in the Results (Tables S6–S11). While several studies report improvements in fibre digestibility, others show limited or non-significant effects [9,10,17]. This inconsistency indicates that the effects of clay supplementation on digestibility are not uniform and may depend on mineral type, composition, inclusion level, and basal diet characteristics [6,12]. Residual heterogeneity may also reflect differences in forage-to-concentrate ratio, clay mineral purity, geological origin, feeding duration, adaptation period, analytical methodology, and animal production stage, which could not be fully accounted for in the moderator analyses. In particular, excessive inclusion levels may reduce digestibility, indicating that optimal dosing is critical to maximise benefits. Unlike previous meta-analyses that primarily evaluated tannins, essential oils, chemical inhibitors, or broader nutritional methane-mitigation strategies [8,20,21,22,23,24,25,26], the present study specifically focused on clay and clay mineral supplementation while simultaneously evaluating fermentation, digestibility, health, and productivity outcomes. This integrated assessment provides a broader understanding of the environmental and production-related implications of clay supplementation and identifies key factors contributing to response variability across studies. This is consistent with recent quantitative syntheses integrating nutrient digestibility, rumen fermentation, blood metabolites, and productive performance in animal nutrition [51].

Health-related responses support the rumen-specific mode of action of clays. Studies have reported reductions in blood urea nitrogen following clay supplementation, which may indicate improved nitrogen utilisation and reduced nitrogen losses [15,16]. However, this response may also reflect differences in dietary protein supply, hepatic urea metabolism, or ruminal ammonia absorption. This finding is consistent with improved nitrogen recycling within the rumen system and enhanced microbial capture and utilisation of ammonia nitrogen. Such changes could promote microbial protein synthesis rather than urea excretion. Previous studies have also reported that lipid profiles and other systemic biomarkers are often unaffected. This confirms that the effects of clays are predominantly localised within the rumen environment. This distinction is important, as it indicates that clays function as physicochemical regulators of rumen processes rather than metabolic modifiers at the host level [10,15,52]. Productivity responses are consistent with these rumen-level improvements. Antonelo, et al. [53] and Maki, et al. [54] reported improved feed efficiency and animal performance following clay supplementation, while Ortiz, et al. [17] observed enhanced weight gain in ruminants receiving clay-based additives. Collectively, these findings suggest that improvements in rumen fermentation and nutrient utilisation may translate into favourable productive responses under clay supplementation. These improvements may be associated with reduced methane losses and improved nutrient utilisation. However, because enteric methane typically accounts for approximately 2–12% of gross energy intake, improvements in productivity are likely multifactorial and cannot be attributed solely to reduced methane emissions [26].

From a mechanistic perspective, clays act as multifunctional rumen modulators. They can adsorb toxins and microbial metabolites [52], buffer rumen pH [10], and influence microbial populations [1,11], including protozoa, bacteria, and archaea [15]. In addition, they may redirect hydrogen utilisation towards propionate formation, thereby reducing methane production while maintaining fermentation efficiency [9,43,44]. These mechanisms have been proposed in previous in vitro and in vivo studies. Potential mechanisms may involve adsorption, elemental dissolution, and cation exchange during clay–microbe interactions [1,11]. However, these mechanisms were not directly evaluated in the present meta-analysis and should therefore be interpreted as plausible explanations rather than direct findings of this study.

From a practical perspective, clay supplementation represents a relatively accessible nutritional strategy that may contribute to methane mitigation while maintaining animal productivity. The effectiveness of this approach is likely to depend on clay mineral type, supplementation level, diet composition, and production system. Intensive production systems may benefit from greater control of supplementation rates and dietary formulation, whereas smallholder systems may benefit from the affordability and local availability of naturally occurring clay minerals. The observed reductions in methane emissions also support the potential inclusion of clay supplementation within broader livestock greenhouse-gas mitigation strategies. However, widespread implementation will require careful consideration of economic feasibility, regulatory approval across different jurisdictions, consistency in clay composition between commercial sources, rigorous quality control, potential mineral contaminants such as heavy metals, long-term safety, and the environmental sustainability of clay extraction, together with validation under commercial production conditions.

## 5. Limitations of the Study

Several limitations should be considered when interpreting these meta-data findings. First, the literature search was restricted to a ten-year window (2015–2025) and limited to PubMed and Scopus, which may exclude which may have excluded relevant studies indexed exclusively in other databases such as Web of Science, CAB Abstracts, or AGRICOLA, as well as industry reports and patent repositories. In addition, only English-language publications were included, which may have excluded relevant studies published in other languages and introduced language bias. Substantial methodological heterogeneity was observed across studies, including that related to animal species, diet composition, and mineral formulation. In addition, key physicochemical properties of these minerals such as particle size, surface area, and mineral composition were often not reported. These factors may constraint in-depth mechanistic interpretation. Inconsistent terminology, particularly the use of “nano” without standardised particle-size characterisation, further complicates cross-study comparisons and may introduce classification bias. Most studies were conducted under short-term or controlled conditions, which may not fully reflect long-term responses in commercial production systems. Furthermore, the quality assessment was based primarily on reported methodological characteristics, and incomplete reporting in some studies limited the evaluation of potential sources of bias. Although a certainty-of-evidence assessment was conducted, confidence in several outcome domains was reduced by between-study heterogeneity and evidence of publication bias. These limitations highlight the need for standardised reporting practices, improved integration of industry-relevant data, comprehensive characterisation of clay mineral properties, and long-term evaluations to strengthen the reliability and applicability of future research.

## 6. Conclusions and Future Outlook

This meta-analysis showed that clay supplementation was associated with significant changes in rumen fermentation, digestibility, animal health, and productivity outcomes. Based on the available evidence, low (<10 g/kg DM), medium (10–20 g/kg DM), and high (>20 g/kg DM) inclusion levels were associated with variable responses depending on clay type, animal species, diet composition, and experimental conditions. Therefore, optimisation of supplementation level should be considered to maximise methane mitigation while maintaining animal health and productivity. The observed reductions in methane production, improvements in fibre digestibility, enhanced nitrogen utilisation, and positive productivity responses are consistent with mechanisms proposed in previous studies. The findings suggest that clay supplementation is associated with improved fermentation efficiency and nutrient utilisation while maintaining overall rumen stability. Enhanced fibre digestibility and nutrient utilisation are consistent with improved microbial colonisation and substrate degradation proposed in previous studies. These may contribute to improved animal performance, including growth and milk component synthesis. The observed improvements in nitrogen utilisation and fermentation stability were associated with animal health outcomes, highlighting the potential dual benefits of clay supplementation at both microbial and host levels. However, responses are not uniform and depend on mineral characteristics, inclusion level, animal species, dietary composition, and dietary conditions, reflecting the complexity of clay–microbe–substrate interactions. Therefore, optimal supplementation strategies should account for these factors, which contribute to variability in response.. In addition, limitations related to material characterisation, restricted literature scope, exclusion of industry and patent data, and the predominance of short-term studies should be considered when interpreting these findings. Overall, the available evidence suggests that clay minerals have considerable potential as multifunctional feed additives associated with improved rumen efficiency and animal productivity. However, their effectiveness depends on clay type, inclusion level, animal species, diet composition, and experimental conditions. Reduced methane production may conserve dietary energy that could potentially be redirected towards improved animal health and productivity. Future research should prioritise long-term in vivo evaluations, with special attention to dose–response frameworks of particular mineral type in the clay or zeolite groups. Such studies would improve confidence in data-driven outcomes and support practical application of these materials in animal feeding strategies.

## Figures and Tables

**Table 1:** Effects of clay supplementation on ruminal fermentation parameters *.

Outcomes	Units	Effect Sizes	Relative Effects (%)	95% Confidence Intervals		*p*-Value
				Lower Values	Upper Values	
Total Gas	mL/g DM	4	−10.28	−26.08	8.90	0.272
CH_4_	mL/g DM	9	−20.45	−29.38	−10.38	<0.001
pH	-	39	0.25	−1.30	1.83	0.753
Total VFA	mmol/L	44	3.25	−1.71	8.46	0.202
Acetate	mmol/L	44	1.58	−2.49	5.82	0.453
Propionate	mmol/L	44	4.35	0.07	8.82	0.046
Butyrate	mmol/L	44	3.11	−4.39	11.20	0.426
Isobutyrate	mmol/L	29	2.94	−6.53	13.36	0.556
Valerate	mmol/L	36	−0.89	−9.55	8.60	0.848
Isovalerate	mmol/L	30	1.75	−6.32	10.50	0.681
NH_3_-N	mmol/L	23	−11.51	−20.91	−1.00	0.033
Protozoa	×10^5^ cells/mL	15	3.45	−12.43	22.22	0.690

Note: * This is the overall effects relative to control. Here, effect sizes represent the number of studies; VFA, Volatile fatty acid; NH_3_-N, Ammonia nitrogen. Values represent pooled percentage change relative to control with corresponding 95% confidence intervals and *p*-values. Positive values indicate an increase, whereas negative values indicate a reduction in the measured parameter following clay supplementation.

**Table 2:** Effects of clay supplementation on health-related parameters across studies.

Outcomes	Units	Effect Sizes	Relative Effects (%)	95% Confidence Intervals		*p*-Value
				Lower Value	Upper Value	
Total protein	g/dL	23	−0.81	−2.75	1.17	0.418
Albumin	g/dL	24	1.11	−2.16	4.49	0.510
Globulin	g/dL	15	−1.68	−8.33	5.46	0.636
Urea	mg/dL	15	−1.32	−6.46	4.09	0.625
Glucose	mg/dL	15	2.93	−3.05	9.28	0.344
ALT	U/L	18	1.37	−4.37	7.46	0.647
AST	U/L	17	−0.21	−8.12	8.40	0.961
Creatinine	mg/d	12	−4.52	−8.91	0.08	0.053
Bilirubin	mg/d	4	−4.28	−34.58	40.05	0.821
BUN	mg/d	6	−5.64	−8.97	−2.18	0.002
TG	mg/d	10	−6.28	−16.24	4.85	0.257
TC	mg/d	12	−2.76	−7.97	2.74	0.318
HDL	mg/d	4	4.43	−10.61	22.01	0.585
Creatine kinase	U/L	4	−0.59	−12.58	13.05	0.929
Calcium	mg/dL	9	5.22	−1.55	12.44	0.134
Sodium	mmol/L	4	−1.33	−7.44	5.18	0.681
Potassium	mmol/L	4	0.66	−8.80	11.11	0.896
Chloride	mmol/L	4	−1.70	−5.36	2.11	0.377

Note: Effect sizes represent the number of studies; ALT, Alanine Aminotransferase; AST, Aspartate Aminotransferase; BUN, Blood Urea Nitrogen; HDL, High-Density Lipoprotein; TC, Total Cholesterol; TG, Triglycerides. Values represent percentage change relative to control with corresponding 95% confidence intervals and *p*-values. Positive values indicate an increase, whereas negative values indicate a reduction in the measured parameter following clay supplementation.

**Table 3:** Publication bias assessment across outcome domains.

Domain	Outcome	*n*	k	Egger z	Egger *p*-Values	Trimfill Added
Fermentation	Methane (CH_4_)	7	85	1.83	0.067	0
	Ammonia Nitrogen (NH_3_-N)	21	187	4.32	<0.001	0
	Propionate	34	349	4.35	<0.001	0
Digestibility	Dry matter	22	136	1.60	0.109	0
	Organic matter	26	140	7.71	<0.001	0
	Neutral detergent fibre	23	127	−0.45	0.653	40
	Nitrogen free extract	6	38	−4.64	<0.001	0
Health	Blood Urea Nitrogen (BUN)	6	54	1.14	0.256	2
Productivity	Weight gain	29	57	2.20	0.028	6
	Fat yield	14	104	2.66	0.008	8
	Lactose yield	13	112	3.38	<0.001	5

Note: *n*, number of studies; k, effect sizes; z, Egger’s regression test.

**Table 4:** Sensitivity analysis results across outcome domains.

Domain	Outcome	*n*	LOO Percent	LOO Range	Max Change	Effect
Fermentation	CH_4_	7	−23.67	−25.65 to −20.69	2.98	No
	NH_3_-N	21	−15.59	−17.78 to −11.01	4.58	No
	Propionate	34	5.48	5.10 to 5.90	0.42	No
Digestibility	Dry matter	22	3.54	3.28 to 3.81	0.27	No
	Organic matter	26	3.15	2.97 to 3.32	0.17	No
	Neutral detergent fibre	23	8.70	7.37 to 9.48	1.32	No
	Nitrogen free extract	6	2.04	1.31 to 2.23	0.73	No
Health	BUN	6	−5.62	−8.10 to −3.08	2.54	No
Productivity	Weight gain	29	7.12	6.39 to 7.60	0.73	No
	Fat yield	14	12.27	9.56 to 15.44	3.16	No
	Lactose yield	13	5.88	3.94 to 7.29	1.94	No

Note: *n*, number of studies from control and treatment groups; LOO, Leave-one-out.

**Table 5:** Comparison of enteric methane reduction by major dietary feed additives.

Feed Additive	Approximate CH_4_ Reduction	Evidence Source
Macroalgae (*Asparagopsis* spp.)	51%	[25]
3-Nitrooxypropanol (3-NOP)	31%	[25]
Nitrate	16%	[25]
Essential oils/plant oils	15%	[25]
Phytochemicals (including tannins)	14%	[25]
Clay minerals (present study)	20.45%	Present meta-analysis

Note: Values are approximate and compiled from published meta-analyses.

## Data Availability

The underlying datasets including supplementary materials supporting this meta-analysis have been deposited in Zenodo and are publicly available at https://doi.org/10.5281/zenodo.19600870.

## Supplementary Materials

The following supporting information can be downloaded at: https://www.mdpi.com/article/10.3390/ani16172646/s1, Supplementary materials supporting this meta-analysis have been deposited in Zenodo and are publicly available at https://doi.org/10.5281/zenodo.19600870. Checklist S1. PRISMA 2020 checklist used to guide the reporting of this systematic review and meta-analysis. Figure S1. PubMed search strategy conducted on 19 November 2025. Figure S2. Scopus search strategy conducted on 19 November 2025. Table S1. Descriptive characteristics of the studies. Table S2. A summary of the study quality assessment. Table S3. Composition of clays and clay minerals across the treatment group. Table S4. Dietary composition across the studies (g/kg DM). Table S5. Summary of analytical methodologies used to quantify fermentation, digestibility, health, and productivity outcomes across included studies. Table S6. Subgroup analysis of the effects of dietary clay supplementation on rumen fermentation parameters in in vivo and in vitro studies. Table S7. Effects of influencing factors other than control and treatment groups on rumen fermentation parameters. Table S8. Subgroup analysis of the effects of dietary clay supplementation on nutrient digestibility in in vivo and in vitro studies. Table S9. Effects of influencing factors other than control and treatment groups on ruminal digestibility. Table S10. Impacts of influencing factors other than control and treatment groups on ruminant health indicators. Table S11. Effects of factors other than control and treatment groups on ruminant health indicators and productivity. Table S12. Assessment of certainty of evidence using GRADE domains.

## Abbreviations

The following abbreviations are used in this manuscript:

ADF	Acid detergent fiber
β	estimated regression coefficient
BUN	Blood urea nitrogen
CEC	Cation exchange capacity
CF	Crude fiber
CH_4_	Methane
CI	Confidence interval
CP	Crude protein
DM	Dry matter
DMI	Dry matter intake
EE	Ether extract
I^2^	Heterogeneity statistic
k	Number of study or effect sizes
LOO	Leave-one-out
lnRR	Log response ratio
NDF	Neutral detergent fiber
NFE	Nitrogen-free extract
NH_3_-N	Ammonia nitrogen
OM	Organic matter
PRISMA	Preferred Reporting Items for Systematic Reviews and Meta-Analyses
REML	Restricted maximum likelihood
RR	Response ratio
SD	Standard deviation
SEM	Standard error of the mean
TC	Total cholesterol
TG	Triglycerides
τ^2^	Between-study variance
VFA	Volatile fatty acids
