# Supplementary material for: Clays as Additives in Ruminant Feed: A Quantitative Meta-Analysis of Their Effects on Enteric Methane, Digestion, Health and Productivity
Source: Animals (Basel). 2026 Aug 24;16(17):2646. doi: 10.3390/ani16172646 (PMC13564275; doi:10.3390/ani16172646)
Supplement: Supplementary file 1 [file animals-16-02646-s001.zip › animals-4426020-supplementary.pdf]

[The headings here correspond to those used in the main manuscript]

2. Materials and Methods

2.1 Literature search and study selection

PRISMA 2020 Checklist

Section and Topic	Item #	Checklist item	Location where item is reported
**TITLE**			
Title	1	Identify the report as a systematic review.	p.1
**ABSTRACT**			
Abstract	2	See the PRISMA 2020 for Abstracts checklist. Sec.2.2 ; Tab.S2	p.1
**INTRODUCTION**			
Rationale	3	Describe the rationale for the review in the context of existing knowledge.	pp. 2-3
Objectives	4	Provide an explicit statement of the objective(s) or question(s) the review addresses.	p.3
**METHODS**			
Eligibility criteria	5	Specify the inclusion and exclusion criteria for the review and how studies were grouped for the syntheses.	pp. 3-4
Information sources	6	Specify all databases, registers, websites, organisations, reference lists and other sources searched or consulted to identify studies. Specify the date when each source was last searched or consulted.	p.3
Search strategy	7	Present the full search strategies for all databases, registers and websites, including any filters and limits used.	p.3 and SI
Selection process	8	Specify the methods used to decide whether a study met the inclusion criteria of the review, including how many reviewers screened each record and each report retrieved, whether they worked independently, and if applicable, details of automation tools used in the process.	pp. 3-4
Data collection process	9	Specify the methods used to collect data from reports, including how many reviewers collected data from each report, whether they worked independently, any processes for obtaining or confirming data from study investigators, and if applicable, details of automation tools used in the process.	p.4
Data items	10a	List and define all outcomes for which data were sought. Specify whether all results that were compatible with each outcome domain in each study were sought (e.g. for all measures, time points, analyses), and if not, the methods used to decide which results to collect.	pp. 4-5
	10b	List and define all other variables for which data were sought (e.g. participant and intervention characteristics, funding sources). Describe any assumptions made about any missing or unclear information.	pp. 4-5
Study risk of bias assessment	11	Specify the methods used to assess risk of bias in the included studies, including details of the tool(s) used, how many reviewers assessed each study and whether they worked independently, and if applicable, details of automation tools used in the process.	Sec.2.2 ; Tab.S2
Effect measures	12	Specify for each outcome the effect measure(s) (e.g. risk ratio, mean difference) used in the synthesis or presentation of results.	pp. 5–6
Synthesis methods	13a	Describe the processes used to decide which studies were eligible for each synthesis (e.g. tabulating the study intervention characteristics and comparing against the planned groups for each synthesis (item #5)).	pp. 3–5
	13b	Describe any methods required to prepare the data for presentation or synthesis, such as handling of missing summary statistics, or data conversions.	p.4
	13c	Describe any methods used to tabulate or visually display results of individual studies and syntheses.	Fig.1-4; Tab.1-3
	13d	Describe any methods used to synthesize results and provide a rationale for the choice(s). If meta-analysis was performed, describe the model(s), method(s) to identify the presence and extent of statistical heterogeneity, and software package(s) used.	p.6
	13e	Describe any methods used to explore possible causes of heterogeneity among study results (e.g. subgroup analysis, meta-regression).	Sec.2.4, 2.6, 3.1, 3.4
	13f	Describe any sensitivity analyses conducted to assess robustness of the synthesized results.	pp.6-7
Reporting bias assessment	14	Describe any methods used to assess risk of bias due to missing results in a synthesis (arising from reporting biases).	pp.6-7
Certainty assessment	15	Describe any methods used to assess certainty (or confidence) in the body of evidence for an outcome.	Section 2.3
**RESULTS**			
Study selection	16a	Describe the results of the search and selection process, from the number of records identified in the search to the number of studies included in the review, ideally using a flow diagram.	pp. 3–4
	16b	Cite studies that might appear to meet the inclusion criteria, but which were excluded, and explain why they were excluded.	p.3
Study characteristics	17	Cite each included study and present its characteristics.	Tab.S1; Fig.2; Sec.2.2
Risk of bias in studies	18	Present assessments of risk of bias for each included study.	Sec.2.2 ; Tab.S2
Results of individual studies	19	For all outcomes, present, for each study: (a) summary statistics for each group (where appropriate) and (b) an effect estimate and its precision (e.g. confidence/credible interval), ideally using structured tables or plots.	Tab.1–2; Fig.3, 4; Tab.S1–S4
Results of syntheses	20a	For each synthesis, briefly summarise the characteristics and risk of bias among contributing studies.	Tab. S1
	20b	Present results of all statistical syntheses conducted. If meta-analysis was done, present for each the summary estimate and its precision (e.g. confidence/credible interval) and measures of statistical heterogeneity. If comparing groups, describe the direction of the effect.	Sec.3.1,3.4; Tab.1,2; Fig.3,4
	20c	Present results of all investigations of possible causes of heterogeneity among study results.	Sec.3.1,3.4; Tab.S5,S8
	20d	Present results of all sensitivity analyses conducted to assess the robustness of the synthesized results.	Sec.3.5; Tab.3
Reporting biases	21	Present assessments of risk of bias due to missing results (arising from reporting biases) for each synthesis assessed.	Sec.2.7, 3.5; Tab.2
Certainty of evidence	22	Present assessments of certainty (or confidence) in the body of evidence for each outcome assessed.	Sec.3.6, Tab.S10
**DISCUSSION**			
Discussion	23a	Provide a general interpretation of the results in the context of other evidence.	pp. 12–13
	23b	Discuss any limitations of the evidence included in the review.	pp. 13–14
	23c	Discuss any limitations of the review processes used.	pp. 13–14
	23d	Discuss implications of the results for practice, policy, and future research.	pp. 14
**OTHER INFORMATION**			
Registration and protocol	24a	Provide registration information for the review, including register name and registration number, or state that the review was not registered.	Not registered
	24b	Indicate where the review protocol can be accessed, or state that a protocol was not prepared.	No protocol prepared
	24c	Describe and explain any amendments to information provided at registration or in the protocol.	Not applicable
Support	25	Describe sources of financial or non-financial support for the review, and the role of the funders or sponsors in the review.	p. 14
Competing interests	26	Declare any competing interests of review authors.	p. 14
Availability of data, code and other materials	27	Report which of the following are publicly available and where they can be found: template data collection forms; data extracted from included studies; data used for all analyses; analytic code; any other materials used in the review.	p. 14

From: Page MJ, McKenzie JE, Bossuyt PM, Boutron I, Hoffmann TC, Mulrow CD, et al. The PRISMA 2020 statement: an updated guideline for reporting systematic reviews. BMJ 2021;372:n71. doi: 10.1136/bmj.n71. This work is licensed under CC BY 4.0. To view a copy of this license, visit https://creativecommons.org/licenses/by/4.0/

Checklist S1. PRISMA 2020 checklist used to guide the reporting of this systematic review and meta–analysis.

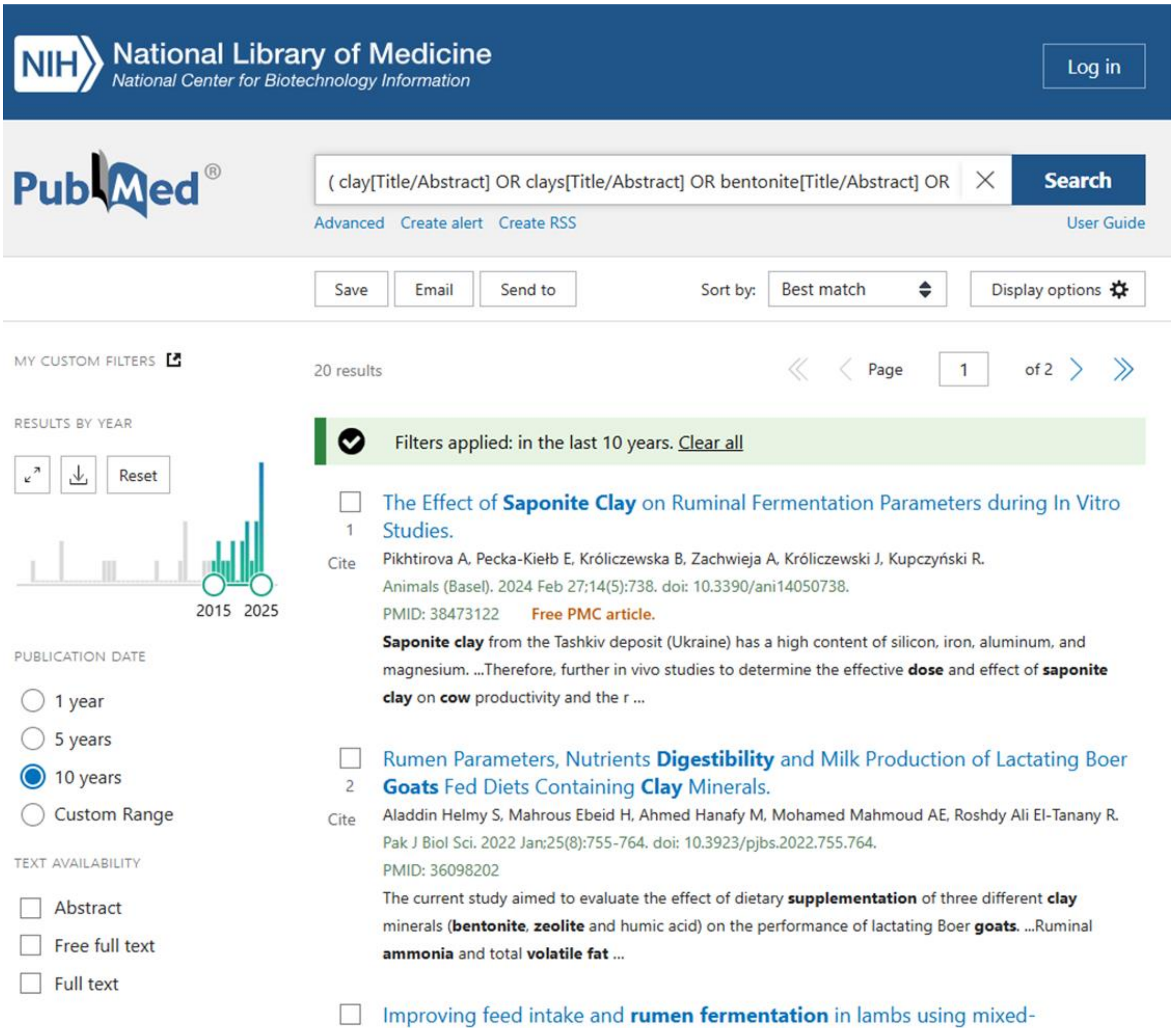

Figure S1. PubMed search conducted 19 November 2025 yielding 20 records. Complete Boolean search string: (clay[Title/Abstract] OR clays[Title/Abstract] OR bentonite[Title/Abstract] OR kaolin[Title/Abstract] OR montmorillonite[Title/Abstract] OR zeolite[Title/Abstract] OR saponite[Title/Abstract] OR sepiolite[Title/Abstract]) AND (cattle[Title/Abstract] OR cow[Title/Abstract] OR bovine[Title/Abstract] OR ruminant*[Title/Abstract] OR sheep[Title/Abstract] OR goat*[Title/Abstract] OR lamb[Title/Abstract] OR buffalo[Title/Abstract] OR calves[Title/Abstract]) AND ("feed additive"[Title/Abstract] OR "dietary supplement"[Title/Abstract] OR supplementation[Title/Abstract] OR Inclusion level[Title/Abstract]) AND ("rumen fermentation"[Title/Abstract] OR "rumen health"[Title/Abstract] OR "volatile fatty acids"[Title/Abstract] OR pH[Title/Abstract] OR ammonia[Title/Abstract] OR methane[Title/Abstract] OR "digestibility"[Title/Abstract] OR "animal productivity"[Title/Abstract] OR "livestock performance"[Title/Abstract]).

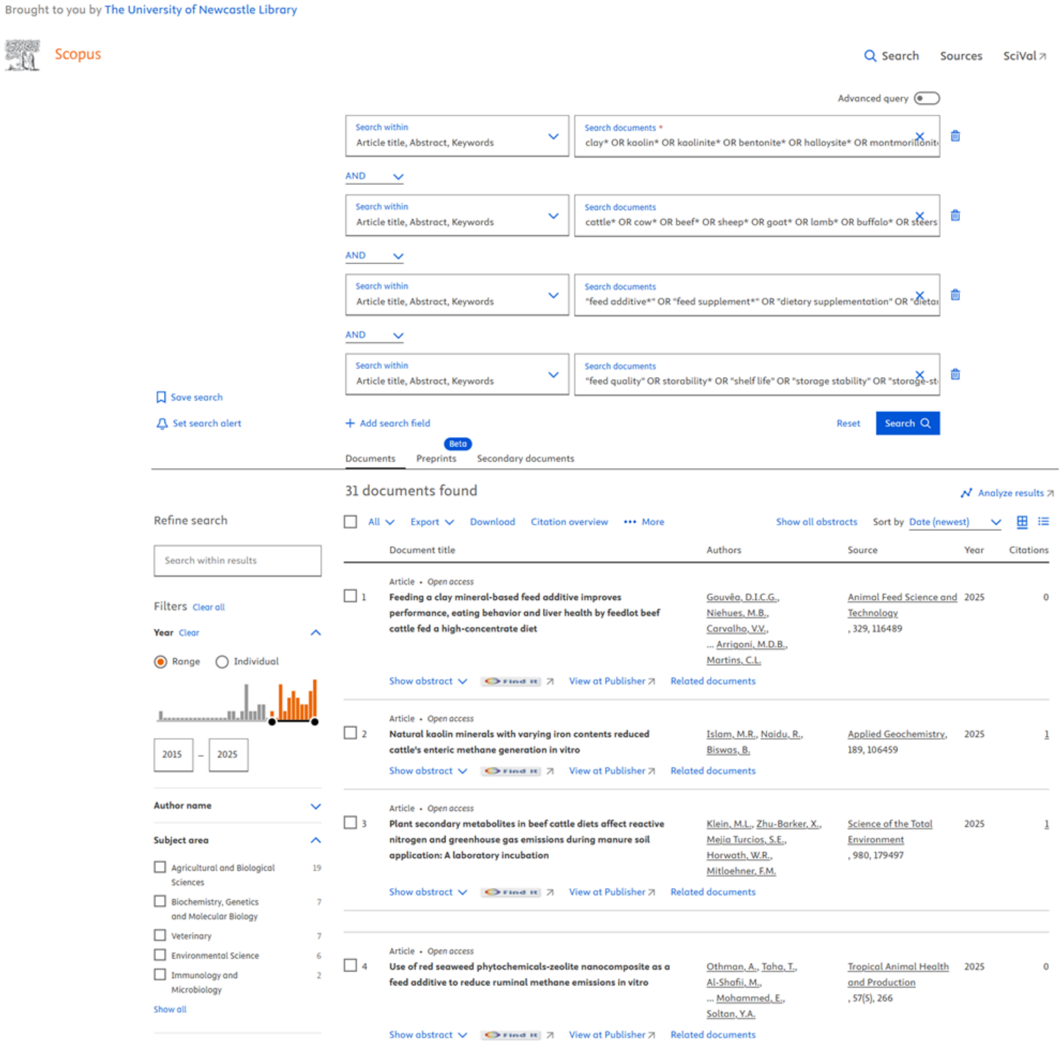

Figure S2. Scopus search conducted 19 November 2025 yielding 31 records. Complete Boolean search string: (TITLE–ABS–KEY(clay* OR kaolin* OR kaolinite* OR bentonite* OR halloysite* OR montmorillonite* OR zeolite* OR saponite* OR sepiolite* OR smectite* OR "clay minerals*")) AND (TITLE–ABS–KEY(cattle* OR cow* OR beef* OR sheep* OR goat* OR lamb* OR buffalo* OR steers* OR Holstein* OR calves* OR ewes*)) AND (TITLE–ABS–KEY("feed additive*" OR "feed supplement*" OR "dietary supplementation" OR "dietary supplement*" OR "dietary additive*")) AND (TITLE–ABS–KEY("feed quality" OR storability* OR "shelf life" OR "storage stability" OR "storage–stability" OR "animal productivity" OR "livestock performance" OR health OR methane OR microbe* OR fermentation* OR digestion*)).

Table S1. Descriptive characteristics of the studies

N	Study/Citations	No. of treat.	Species	Exp. types	Clay (& zeolite) types	Inclusion levels g/kg DM	Exp. Duration
1.	Al Adawi, *et al.* [1]	2	Dairy Cow	*in vivo*	Nano montmorillonite	1.5 and 3	21 d
1.	Alrez, *et al.* [2]	1	Lamb	*in vivo*	Zeolite	30	15 d
2.	Antonelo, *et al.* [3]	2	Beef	*in vivo*	Mg and Fe−rich clay minerals	10 and 20	21 and 33 d
3.	Azadbakht, *et al.* [4]	2	Lamb	*in vivo* and *in vitro*	Bentonite	15 and 20	70 d and 96 h
4.	Damato, *et al.* [5]	4	Dairy Cow	*in vitro*	Bentonite	2.5, 5, 10 and 50	24 h
5.	El-Nile, *et al.* [6]	3 and 5	Goat	*in vitro* and *in vivo*	Zeolite and Nano−zeolite	10, 20, 30 and 0.2, 0.3, 0.4, 0.5, 1.0	24 h
6.	El-Nile, *et al.* [7]	2	Dairy Cow	*in vivo*	Zeolite and Nano−zeolite	20 and 0.4	90 d
7.	Aladdin Helmy, *et al.* [8]	2	Goat	*in vivo*	Bentonite and Zeolite	10 and 12.5	45 d
8.	Islam, *et al.* [9]	2	Beef	*in vitro*	Kaolin and Iron−rich Kaolin	50 and 50	48 h
9.	Jiang, *et al.* [10]	2	Dairy Cow	*in vivo*	Bentonite	6.52	33 da
10.	Myers, *et al.* [11]	1	Beef	*in vivo*	Zeolite	25	21 d
11.	Othman, *et al.* [12]	1	Buffalo	*in vitro*	Zeolite	20	12 h
12.	Urías-Estrada, *et al.* [13]	1	Lamb	*in vivo*	Zeolite	30	34 d
13.	Zeltwanger, *et al.* [14]	1	Beef	*in vivo*	Zeolite	2	21 d
14.	Amanzougarene and Fondevila [15]	3	Ewe	*in vitro*	Zeolite, bentonite, and sepiolite	10,10 and 10	24 h
15.	Schlattl, *et al.* [16]	1	Non−dairy Cow	*in vitro*	Bentonite	0.2	24 h
16.	Abo-Sherif, *et al.* [17]	1	Non−dairy Cow	*in vitro*	Bentonite	5	48 h
17.	Pikhtirova, *et al.* [18]	2	Non−dairy Cow	*in vitro*	Saponite	37.5 and 62.5	24 h
18.	Chen, *et al.* [19]	3	Lamb	*in vivo*	Mg−rich aluminosilicate	0.5, 1.0, and 2.0	56 d
19.	Sulzberger, *et al.* [20]	3	Non−dairy Cow	*in vivo*	Mg and Fe−rich clay minerals	5, 10 and 20	21 d
20.	Ortiz, *et al.* [21]	2	Beef	*in vivo*	Kaolinite	10 and 20	308 d
21.	Gouda, *et al.* [22]	2	Goat	*in vivo*	Bentonite and Montmorillonite	20 and 20	90 d
22.	Soltan, *et al.* [23]	1	Buffalo	*in vitro*	Nano−montmorillonite	5	24 h

23.	Hussein, *et al.* [24]	1	Buffalo	*in vivo*	Bentonite	10	14 d
24.	Fabino Neto, *et al.* [25]	3	Lamb	*in vivo*	Natural clay−based micromineral compound	4, 8, and 12	90 d
25.	Tate, *et al.* [26]	2	Non−dairy Cow	*in vitro*	Kaolinite	3.75 and 7.5	7 h
26.	Gallo, *et al.* [27]	1	Dairy Cow	*in vivo*	Bentonite	5.43	21 d
27.	Mehany and Shams [28]	3	Dairy Cow	*in vivo*	Bentonite, Bentonite−zeolite mix, and Zeolite	20, 20 and 20	180
28.	Burçak and Yalçın [29]	2	Lamb	*in vivo*	Sepiolite	10 and 20	90 d
29.	Razavi, *et al.* [30]	1	Dairy Cow	*in vivo*	Bentonite	6.52	7 w
30.	Kihal, *et al.* [31]	1	Dairy Cow	*in vivo*	Montmorillonite	12	48 h
31.	Biswas, *et al.* [32]	3	Beef	*in vitro*	illite	5, 10 and 20	24 h
32.	Zhang, *et al.* [33]	1	Dairy Cow	*in vitro*	Nano−montmorillonite	1	120 m
33.	Maki, *et al.* [34]	2	Dairy Cow	*in vivo*	Montmorillonite	5 and 10	14 d
34.	Castro-Pérez, *et al.* [35]	3	Lamb	*in vivo*	Zeolite (60), Zeolite (100) and Zeolite (200)	30, 30 and 30	67 d
35.	Maki, *et al.* [36]	2	Dairy Cow	*in vivo*	Montmorillonite	1.25 and 2.5	10 d
36.	Aydın, *et al.* [37]	2	Lamb	*in vivo*	Bentonite	10 and 20	45 d
37.	Ismail, *et al.* [38]	1	Sheep	*in vitro*	Bentonite	40	24 h
38.	Roque-Jiménez, *et al.* [39]	3	Ewe	*in vivo*	Zeolite	20, 40 and 60	52 d

Note: N, Number of selected papers; DM, Dry matter; w, weeks; d, days; h, hours; m, minutes.

2.2 Study Quality Assessment

Table S2. A summary of the study quality assessment

Assessment domain	Low risk, n (%)	Some concerns, n (%)	High risk, n (%)
Appropriate control group	39 (100)	0 (0)	0 (0)
Randomisation of animals/experimental units	24 (61.5)	15 (38.5)	0 (0)
Replication/sample adequacy	31 (79.5)	8 (20.5)	0 (0)
Variance reporting (SD or SEM)	39 (100)	0 (0)	0 (0)

Note: Study quality was assessed based on reported methodological characteristics. Because study protocols were not available for most publications, selective outcome reporting could not be reliably assessed and was therefore not included in the summary assessment.

Abbreviations

ADF — Acid detergent fiber
β — estimated regression coefficient.
BUN — Blood urea nitrogen
CEC — Cation exchange capacity
CF — Crude fiber
CH_4 — Methane
CI — Confidence interval
CP — Crude protein
DM — Dry matter
DMI — Dry matter intake
EE — Ether extract
I^2 — Heterogeneity statistic
k — Number of study or effect sizes
LOO — Leave–one–out
lnRR — Log response ratio
NDF — Neutral detergent fiber
NFE — Nitrogen–free extract
NH_3–N — Ammonia nitrogen
OM — Organic matter
PRISMA — Preferred Reporting Items for Systematic Reviews and Meta–Analyses
REML — Restricted maximum likelihood
RR — Response ratio
SD — Standard deviation
SEM — Standard error of the mean
TC — Total cholesterol
TG — Triglycerides
τ^2 — Between–study variance
VFA — Volatile fatty acids

2.4 Data extraction and outcome classification

Table S3. Composition of clays and clay minerals across the treatment group.

Elements	Zeolite[1,2] (n=2)	Kaolin[3,4] (n=2)	Bentonite[5,6] (n=2)	Clay*[7] (n=1)	Sepiolite[8] (n=1)	Illite[9] (n=1)
SiO_2	68.33 ± 0.25	55.59 ± 1.68	58.88 ± 2.85	51.84	41.8	66.14
Al_2O_3	11.65 ± 0.92	38.72 ± 2.57	17.62 ± 0.16	12.53	1.1	23.04
Fe_2O_3	1.33 ± 0.04	2.16 ± 2.24	7.87 ± 5.70	13.88	0.5	Trace
MgO	0.98 ± 0.11	–	2.58 ± 1.04	6.21	21.22	Trace
CaO	3.88 ± 0.11	–	2.60 ± 0.35	9.72	13.5	Trace
K_2O	2.85 ± 0.07	1.98 ± 2.27	0.97 ± 0.03	0.96	0.34	Trace
TiO_2	0.2	0.49 ± 0.25	2.84	1.72	–	Trace
Na_2O	0.61 ± 0.19	–	1.14 ± 1.34	2.2	0.3	Trace
P_2O_5	–	0.52 ± 0.01	–	0.19		Trace
Mn_2O_3	–	–	–	0.21	0.01	–
Cl	–	0.33 ± 0.01	–	–	–	–

Note: Elemental composition data (XRF analysis) were available for a subset of included studies (n = 9 of 39). Values are presented as mean ± standard deviation (SD) calculated from reported weight percentages (%) in the original publications. Given variability in mineral source, geological origin, processing methods, and analytical procedures across studies, these values are summarised descriptively and should be interpreted as indicative rather than standardised compositional profiles. In some cases, SD exceeds the mean value, reflecting substantial between–study heterogeneity rather than analytical error. References corresponding to the contributing studies are provided below.

*Natural clay–based micromineral compound

[1]Alrez, *et al.* [2]

[2]El-Nile, *et al.* [6]

[3,4]Islam, *et al.* [9]

[5]Schlattl, *et al.* [16]

[6]Aydın, *et al.* [37]

[7]Fabino Neto, *et al.* [25]

[8]Burçak and Yalçın [29]

[9]Biswas, *et al.* [32]

Table S4. Dietary composition across the studies (g/kg DM)

Items	Number of studies (n)	Control	Treatment
Ingredients			
Hay	23	314.69 ± 257.29	314.69 ± 257.29
Barley	9	325.98 ± 287.27	321.53 ± 281.35
Wheat straw	9	170.46 ± 280.35	170.46 ± 280.35
Wheat bran	14	251.55 ± 272.53	240.12 ± 275.46
Corn silage	10	212.24 ± 123.34	212.24 ± 123.34
Corn grain	22	352.82 ± 254.09	350.97 ± 250.00
Cons. Feed	9	325.08 ± 317.92	317.96 ± 321.40
Soybean meal	17	124.22 ± 201.60	122.42 ± 202.06
$CaCO_3$	14	11.35 ± 6.86	11.35 ± 6.86
NaCl	13	6.48 ± 6.56	6.48 ± 6.56
Mineral mixture*	25	13.78 ± 13.47	13.74 ± 13.47
Molases	9	54.17 ± 28.66	54.21 ± 28.72
Chemical composition			
Dry matter	20	825.23 ± 138.97	826.30 ± 139.20
Organic matter	15	917.97 ± 42.79	912.52 ± 47.10
Crude protein	32	142.78 ± 42.16	138.17 ± 45.32
Neutral detergent fiber	25	372.03 ± 152.57	371.87 ± 152.37
Acid detergent fiber	19	234.48 ± 123.93	233.85 ± 124.17
Crude fiber	8	100.03 ± 36.02	144.32 ± 114.19
Acid detergent lignin	6	65.80 ± 32.50	65.80 ± 32.50
Non−fiber carbohydrates	10	410.16 ± 271.00	404.56 ± 269.43
Hemicellulose	3	230.23 ± 177.85	230.23 ± 177.85
Cellulose	2	269.00 ± 19.80	269.00 ± 19.80

Note: data were presented as Mean ± SD. In some cases, SD exceeds the mean value, reflecting substantial between−study heterogeneity rather than analytical error.
*Mineral mixture (Ca, P, Se, Vit−A, D, E).

Table S5. Summary of analytical methodologies used to quantify fermentation, digestibility, health, and productivity outcomes across included studies.

Domain	Outcome	Analytical method(s) used in included studies*	Example References
Fermentation	CH_4	Gas chromatography (GC)[1]; Laser gas ditector[2]	[6,18,23][1]; [9,12,17][2]
	VFA	Gas chromatography (GC−FID or GC−TCD)[3]; capillary column systems (e.g., HPLC or HP−FFAP)[4]	[1,9,11][3]; [7,12,15][4]
	NH_3−N	Spectrophotometric[5], Electrode[6]	[7,12,40][5]; [9][6]
Digestibility	DM, OM, and fiber fractions	AOAC method[7]; marker−based digestibility[8]; *in vitro* fiber or digestibility analyser[9]	[1,7,11][7]; [6,28,40][8]; [3,9,15][9]
Health	BUN and other metabolites	Automated clinical biochemical analyzers[10]; spectrophotometric assays[11]	[2,10,25][10]; [3,24,40][11]
Productivity	Weight gain	Standard body weight measurement protocols[12]	[2-4][12]
	Milk yield	Automated milk meters, and weighing to record daily, periodically or monthly volume[13]	[1,7,10][13]
	Milk composition	Automated milk analyser or spectrophotometer[14]	[1,10,27][14]

Note: Analytical approaches were extracted from the methods sections of included studies. Where multiple techniques were used within a domain, all reported methods are summarized. GC, gas chromatography; FID, flame ionization detector; TCD, thermal conductivity detector; AOAC, Association of Official Analytical Chemists.

*Superscripted number represents the associated reference linked to the right column of this table

3. Results

3.1 Effects of Clays Supplementation on Ruminal Fermentation

Table S6. Subgroup analysis of the effects of dietary clay supplementation on rumen fermentation parameters in in vivo and in vitro studies

Outcomes	*in vivo*			*in vitro*		
	Effect sizes (k)	RE (95% CI)	*p*–values	Effect sizes (k)	RE (95% CI)	*p*–values
Total Gas	–	–	–	4	−14.55 (−28.44 to 2.03)	0.082
CH_4	2	−16.25 (−42.64 to 22.28)	0.358	7	−21.94 (−32.96 to −9.10)	< 0.001
pH	23	0.52 (−0.84 to 1.89)	0.457	16	−1.27 (−3.34 to 0.85)	0.239
TVFA	16	6.32 (1.36 to 11.54)	0.012	21	2.59 (−0.82 to 6.11)	0.139
Acetate	23	−1.70 (−3.44 to 0.07)	0.059	21	2.32 (−0.53 to 5.25)	0.112
Propionate	23	2.56 (−2.23 to 7.60)	0.300	21	6.49 (3.73 to 9.33)	< 0.001
Butyrate	23	7.08 (0.27 to 14.36)	0.041	21	−2.62 (−7.79 to 2.84)	0.439
Isobutyrate	15	−4.91 (−13.96 to 5.08)	0.323	14	13.00 (−16.63 to 53.16)	0.431
Valerate	18	−4.22 (−13.68 to 6.27)	0.416	18	−1.06 (−6.07 to 4.21)	0.687
Isovalerate	15	−0.05 (−12.23 to 13.81)	0.994	15	−0.15 (−4.51 to 4.40)	0.946
NH_3–N	9	−15.01 (−27.15 to −0.85)	0.039	14	−15.38 (−28.38 to −0.02)	0.050
Protozoa	6	−3.10 (−13.74 to 8.85)	0.595	9	23.35 (12.05 to 35.80)	< 0.001

Note: TVFA, total volatile fatty acids; NH_3–N, ammonia nitrogen; RE, relative effects in %; CI, confidence interval; *k*, number of effect sizes.

Table S7. Effects of influencing factors other than control and treatment groups on rumen fermentation parameters

Outcome	Influencing factors*	β	SE	z	*p*–values	Relative changes (%)
Acetate	SpeciesCattle	−0.17	0.08	−2.00	0.045	−15.45
Acetate	SpeciesGoat	−0.19	0.11	−1.71	0.088	−16.97
Acetate	SpeciesSheep	−0.19	0.09	−2.13	0.033	−16.92
Acetate	Clay_typeillite	−0.002	0.09	−0.03	0.979	−0.25
Acetate	Clay_typeKaolin	0.03	0.39	0.07	0.944	2.74
Acetate	Clay_typeKaolinite	−0.07	0.10	−0.67	0.500	−6.54
Acetate	Clay_typeNano Montmorillonite	−0.16	0.09	−1.71	0.087	−14.65
Acetate	Clay_typeNano Zeolite	−0.05	0.04	−1.42	0.156	−5.10
Acetate	Clay_typeOthers	−0.02	0.06	−0.42	0.674	−2.46
Acetate	Clay_typeSaponite	0.05	0.10	0.48	0.630	4.71
Acetate	Clay_typeSepiolite	−0.04	0.04	−1.12	0.262	−4.06
Acetate	Clay_typeZeolite	0.01	0.04	0.35	0.729	1.23
Acetate	Inclusion level	−0.002	0.00	−5.10	0.000	−0.21
Butyrate	SpeciesCattle	−0.07	0.11	−0.64	0.524	−6.90
Butyrate	SpeciesGoat	0.00	0.12	−0.03	0.979	−0.32
Butyrate	SpeciesSheep	−0.18	0.12	−1.52	0.130	−16.55
Butyrate	Clay_typeillite	0.11	0.12	0.93	0.353	11.97
Butyrate	Clay_typeKaolin	−0.03	0.22	−0.15	0.883	−3.18
Butyrate	Clay_typeKaolinite	−0.07	0.21	−0.34	0.736	−6.87
Butyrate	Clay_typeNano Montmorillonite	0.04	0.11	0.39	0.697	4.48
Butyrate	Clay_typeNano Zeolite	0.02	0.08	0.31	0.755	2.39
Butyrate	Clay_typeOthers	−0.09	0.09	−0.95	0.341	−8.18
Butyrate	Clay_typeSaponite	−0.47	0.15	−3.18	0.001	−37.70
Butyrate	Clay_typeSepiolite	0.09	0.06	1.36	0.174	8.99
Butyrate	Clay_typeZeolite	0.002	0.06	0.03	0.978	0.18
Butyrate	Inclusion level	0.002	0.00	2.45	0.014	0.18
CH_4	SpeciesCattle	−0.23	0.16	−1.39	0.166	−20.20
CH_4	Clay_typeillite	0.17	0.16	1.02	0.307	18.19
CH_4	Clay_typeSaponite	0.44	0.15	2.96	0.003	54.68
CH_4	Inclusion level	−0.02	0.00	−44.88	0.000	−1.51
Isobutyrate	SpeciesCattle	−0.10	0.61	−0.16	0.874	−9.28
Isobutyrate	SpeciesGoat	−0.01	0.66	−0.02	0.987	−1.03
Isobutyrate	SpeciesSheep	−0.16	0.80	−0.20	0.841	−14.78
Isobutyrate	Clay_typeKaolin	−0.35	0.75	−0.47	0.638	−29.77
Isobutyrate	Clay_typeNano Montmorillonite	0.04	0.83	0.05	0.959	4.35
Isobutyrate	Clay_typeNano Zeolite	0.20	0.76	0.26	0.792	22.24
Isobutyrate	Clay_typeOthers	−0.31	0.74	−0.42	0.678	−26.61
Isobutyrate	Clay_typeSaponite	−0.40	0.78	−0.52	0.605	−33.19
Isobutyrate	Clay_typeSepiolite	−0.24	0.91	−0.27	0.788	−21.70
Isobutyrate	Clay_typeZeolite	−0.43	0.77	−0.56	0.577	−34.99
Isobutyrate	Inclusion level	0.02	0.00	3.68	0.000	1.60

NH_3–N	SpeciesCattle	−0.17	0.27	−0.66	0.511	−16.00
NH_3–N	SpeciesGoat	0.15	0.26	0.56	0.572	15.93
NH_3–N	SpeciesSheep	0.002	0.34	0.01	0.995	0.22
NH_3–N	Clay_typeillite	0.36	0.29	1.26	0.208	43.80
NH_3–N	Clay_typeKaolin	0.06	0.12	0.50	0.614	6.22
NH_3–N	Clay_typeNano Zeolite	0.13	0.10	1.26	0.207	13.55
NH_3–N	Clay_typeOthers	0.08	0.09	0.88	0.380	8.59
NH_3–N	Clay_typeZeolite	0.09	0.09	1.00	0.316	9.68
NH_3–N	Inclusion level	0.01	0.00	3.46	0.001	0.65
pH	SpeciesCattle	0.09	0.06	1.59	0.111	9.44
pH	SpeciesGoat	0.01	0.05	0.10	0.924	0.52
pH	SpeciesSheep	0.10	0.06	1.69	0.091	10.10
pH	Clay_typeillite	−0.12	0.04	−2.89	0.004	−11.05
pH	Clay_typeKaolin	−0.11	0.06	−1.97	0.049	−10.49
pH	Clay_typeKaolinite	−0.07	0.03	−1.92	0.055	−6.30
pH	Clay_typeNano Montmorillonite	−0.06	0.03	−2.03	0.042	−5.95
pH	Clay_typeNano Zeolite	−0.03	0.03	−0.94	0.346	−2.78
pH	Clay_typeOthers	−0.06	0.03	−1.89	0.059	−5.66
pH	Clay_typeSaponite	−0.07	0.03	−2.15	0.031	−7.22
pH	Clay_typeSepiolite	−0.06	0.03	−1.88	0.060	−5.89
pH	Clay_typeZeolite	−0.02	0.03	−0.65	0.513	−1.94
pH	Inclusion level	0.00	0.00	−0.11	0.915	0.00
Propionate	SpeciesCattle	0.01	0.14	0.05	0.956	0.75
Propionate	SpeciesGoat	−0.08	0.17	−0.45	0.654	−7.41
Propionate	SpeciesSheep	0.01	0.15	0.09	0.931	1.30
Propionate	Clay_typeillite	−0.06	0.15	−0.43	0.666	−6.24
Propionate	Clay_typeKaolin	−0.02	0.30	−0.08	0.939	−2.26
Propionate	Clay_typeKaolinite	−0.01	0.17	−0.05	0.961	−0.81
Propionate	Clay_typeNano Montmorillonite	0.02	0.13	0.18	0.859	2.35
Propionate	Clay_typeNano Zeolite	0.08	0.06	1.47	0.140	8.46
Propionate	Clay_typeOthers	−0.03	0.10	−0.27	0.789	−2.57
Propionate	Clay_typeSaponite	0.05	0.15	0.30	0.763	4.75
Propionate	Clay_typeSepiolite	0.06	0.05	1.05	0.294	5.73
Propionate	Clay_typeZeolite	0.01	0.05	0.27	0.788	1.42
Propionate	Inclusion level	0.001	0.00	1.31	0.190	0.10
Protozoa	SpeciesCattle	0.40	0.27	1.46	0.144	49.04
Protozoa	SpeciesGoat	0.19	0.26	0.70	0.483	20.37
Protozoa	Clay_typeNano Zeolite	0.26	0.11	2.39	0.017	29.23
Protozoa	Clay_typeZeolite	0.60	0.12	4.96	0.000	82.74
Protozoa	Inclusion level	−0.02	0.00	−4.76	0.000	−1.58
Total_Gas	SpeciesCattle	0.04	0.06	0.67	0.503	4.19
Total_Gas	Clay_typeSaponite	−0.06	0.05	−1.12	0.263	−5.77
Total_Gas	Inclusion level	−0.01	0.00	−15.76	0.000	−0.64
Total_VFA	SpeciesCattle	−0.01	0.10	−0.05	0.956	−0.55
Total_VFA	SpeciesGoat	−0.04	0.11	−0.34	0.736	−3.52
Total_VFA	SpeciesSheep	−0.07	0.11	−0.70	0.484	−7.12
Total_VFA	Clay_typeillite	−0.04	0.04	−0.91	0.361	−3.58
Total_VFA	Clay_typeKaolin	−0.07	0.07	−1.00	0.319	−6.33

Total_VFA	Clay_typeNano Montmorillonite	−0.05	0.10	−0.55	0.583	−5.26
Total_VFA	Clay_typeNano Zeolite	0.03	0.05	0.75	0.452	3.45
Total_VFA	Clay_typeOthers	−0.07	0.04	−1.77	0.077	−7.03
Total_VFA	Clay_typeSaponite	−0.32	0.07	−4.74	0.000	−27.56
Total_VFA	Clay_typeSepiolite	−0.04	0.04	−1.09	0.277	−4.06
Total_VFA	Clay_typeZeolite	−0.02	0.04	−0.44	0.661	−1.64
Total_VFA	Inclusion level	0.00	0.00	0.12	0.905	0.01
Valerate	SpeciesCattle	0.09	0.17	0.54	0.449	9.53
Valerate	SpeciesGoat	−0.23	0.31	−0.76	0.205	−20.78
Valerate	SpeciesSheep	−0.45	0.35	−1.27	0.476	−36.18
Valerate	Clay_typeKaolin	−0.25	0.35	−0.71	0.125	−21.99
Valerate	Clay_typeNano Montmorillonite	−0.29	0.19	−1.53	0.531	−25.48
Valerate	Clay_typeNano Zeolite	−0.17	0.27	−0.63	0.157	−15.32
Valerate	Clay_typeOthers	−0.13	0.09	−1.41	0.098	−11.93
Valerate	Clay_typeSaponite	−0.29	0.18	−1.65	0.135	−25.20
Valerate	Clay_typeSepiolite	−0.46	0.31	−1.49	0.000	−37.15
Valerate	Clay_typeZeolite	−0.26	0.07	−3.65	0.001	−23.10
Valerate	Inclusion level	−0.25	0.07	−3.43	0.040	−21.86

Note: Estimates represent regression coefficients (β) obtained from multilevel meta−regression models fitted using REML. β, Estimate (log−response ratio, main fixed effect); SE, standard errors; z, Wald z−statistic (β/SE), *p*−values, test of β = 0. VFA, Volatile fatty acids. Relative percentage changes (%) for methane production, ammonia nitrogen (NH_3−N), and VFA profile outcomes from multilevel random−effects meta−analysis, stratified by influencing factors (study design, clay type, dosage, ruminant species). Significant values were highlighted. Inclusion level was analysed as g/kg dry matter (DM). Relative changes (%) represent the predicted change in the outcome per 1 g/kg DM increase in clay supplementation level.

*Variability and its identification are written together as software came up with. Variability is put as red text for visual convenience.

3.2 Influences of Clays on Rumen Digestibility

Table S8. Subgroup analysis of the effects of dietary clay supplementation on nutrient digestibility in in vivo and in vitro studies

Outcomes	*in vivo*			*in vitro*		
	Effect sizes (k)	RE (95% CI)	*p*−values	Effect sizes (k)	RE (95% CI)	*p*−values
DM	23	3.32 (2.01 to 4.65)	< 0.001	–	–	–
OM	23	3.17 (1.89 to 4.47)	< 0.001	3	2.59 (−6.74 to 12.85)	0.368
CP	24	6.07 (0.98 to 11.42)	0.021	–	–	–
EE	17	0.89 (−5.89 to 8.16)	0.790	–	–	–
NDF	20	8.99 (3.65 to 14.61)	0.002	3	6.46 (−24.91 to 50.95)	0.521
ADF	18	18.41 (0.82 to 39.08)	0.041	1	23.19 (−0.37 to 52.31)	0.054
CF	7	3.64 (−3.88 to 11.76)	0.290	–	–	–
NFE	6	2.04 (−0.55 to 4.69)	0.099	–	–	–
Starch	8	−4.30 (−8.50 to 0.09)	0.054	–	–	–

Note: RE, relative effects in %; DM, dry matter; OM, organic matter; CP, crude protein; EE, ether extract; NDF, neutral detergent fibre; ADF, acid detergent fibre; CF, crude fibre; NFE, nitrogen−free extract; RR, relative ratio; CI, confidence interval; k, number of effect sizes.

Table S9. Effects of influencing factors other than control and treatment groups on ruminal digestibility

**Outcome**	**Influencing factors***	**β**	**SE**	**z**	***p*−values**	**Relative changes (%)**
OM	SpeciesCattle	0.04	0.03	1.29	0.197	3.63
OM	SpeciesGoat	0.03	0.03	0.84	0.399	2.74
OM	SpeciesSheep	0.05	0.04	1.26	0.207	5.03
OM	Clay_typeKaolinite	−0.04	0.04	−0.98	0.329	−4.06
OM	Clay_typeMontmorillonite	0.01	0.00	2.80	0.005	0.57
OM	Clay_typeNano Montmorillonite	0.02	0.04	0.54	0.590	2.03
OM	Clay_typeNano Zeolite	−0.06	0.03	−1.78	0.075	−5.87
OM	Clay_typeOthers	−0.01	0.01	−1.38	0.169	−1.45
OM	Clay_typeZeolite	−0.03	0.01	−3.25	0.001	−3.14
OM	Inclusion level	−0.001	0.00	−0.48	0.634	−0.05
CP	SpeciesCattle	0.00	0.15	0.00	0.999	−0.02
CP	SpeciesGoat	0.13	0.15	0.89	0.373	14.28
CP	SpeciesSheep	−0.04	0.15	−0.27	0.784	−4.04
CP	Clay_typeMontmorillonite	0.03	0.01	3.66	0.000	3.20
CP	Clay_typeNano Montmorillonite	−0.05	0.15	−0.33	0.738	−5.03
CP	Clay_typeNano Zeolite	−0.04	0.06	−0.61	0.542	−3.82
CP	Clay_typeOthers	−0.01	0.02	−0.89	0.373	−1.49
CP	Clay_typeZeolite	−0.04	0.02	−2.49	0.013	−3.70
CP	Inclusion level	−0.01	0.00	−7.97	0.000	−0.52
EE	SpeciesCattle	−0.10	0.19	−0.53	0.598	−9.51
EE	SpeciesGoat	−0.21	0.17	−1.22	0.223	−18.94
EE	SpeciesSheep	−0.04	0.18	−0.21	0.830	−3.85
EE	Clay_typeMontmorillonite	0.03	0.01	3.41	0.001	2.62
EE	Clay_typeNano Montmorillonite	−0.11	0.20	−0.57	0.566	−10.79
EE	Clay_typeNano Zeolite	−0.09	0.04	−2.21	0.027	−8.71
EE	Clay_typeOthers	−0.02	0.01	−1.55	0.122	−1.91
EE	Clay_typeZeolite	−0.03	0.01	−2.85	0.004	−3.36
EE	Inclusion level	−0.01	0.00	−18.03	0.000	−0.74
NDF	SpeciesCattle	0.23	0.19	1.19	0.234	25.52
NDF	SpeciesGoat	0.30	0.23	1.30	0.195	35.57
NDF	SpeciesSheep	0.22	0.24	0.94	0.350	24.59
NDF	Clay_typeKaolinite	0.12	0.18	0.67	0.502	12.99
NDF	Clay_typeMontmorillonite	0.04	0.02	2.11	0.035	4.24
NDF	Clay_typeNano Montmorillonite	0.13	0.14	0.92	0.356	13.80
NDF	Clay_typeNano Zeolite	−0.11	0.11	−1.02	0.310	−10.82
NDF	Clay_typeOthers	0.11	0.15	0.76	0.450	11.92
NDF	Clay_typeZeolite	0.01	0.11	0.10	0.924	1.06
NDF	Inclusion level	−0.01	0.00	−4.68	0.000	−0.50
ADF	SpeciesGoat	0.58	0.49	1.20	0.232	78.78
ADF	SpeciesSheep	−0.10	0.54	−0.18	0.855	−9.32

ADF	Clay_typeMontmorillonite	0.02	0.02	0.72	0.469	1.52
ADF	Clay_typeNano Montmorillonite	−0.21	0.68	−0.31	0.757	−18.97
ADF	Clay_typeNano Zeolite	−0.83	0.49	−1.70	0.090	−56.44
ADF	Clay_typeOthers	0.45	0.57	0.79	0.431	56.56
ADF	Clay_typeZeolite	0.13	0.48	0.27	0.786	14.02
ADF	Inclusion level	−0.05	0.00	−9.98	0.000	−4.50
DM	SpeciesCattle	−0.02	0.02	−0.86	0.389	−1.74
DM	SpeciesGoat	−0.03	0.02	−1.38	0.169	−2.74
DM	SpeciesSheep	0.03	0.02	1.29	0.197	3.23
DM	Clay_typeMontmorillonite	0.01	0.00	3.75	0.000	1.06
DM	Clay_typeNano Zeolite	−0.03	0.02	−1.14	0.253	−2.60
DM	Clay_typeOthers	−0.02	0.01	−1.44	0.151	−1.75
DM	Clay_typeZeolite	−0.04	0.01	−3.47	0.001	−3.49
DM	Inclusion level	0.00	0.00	0.44	0.662	0.04
CF	SpeciesCattle	0.99	0.32	3.15	0.002	170.05
CF	SpeciesGoat	0.07	0.05	1.45	0.147	7.58
CF	SpeciesSheep	1.89	0.63	3.00	0.003	564.60
CF	Clay_typeOthers	−0.02	0.02	−1.07	0.287	−2.13
CF	Clay_typeZeolite	−0.04	0.02	−2.09	0.036	−4.17
CF	Inclusion level	−0.10	0.03	−3.07	0.002	−9.30
NFE	SpeciesCattle	0.73	0.22	3.38	0.001	106.83
NFE	SpeciesGoat	0.09	0.04	2.51	0.012	9.40
NFE	Clay_typeOthers	−0.01	0.01	−1.48	0.139	−1.23
NFE	Clay_typeZeolite	−0.02	0.01	−2.25	0.024	−1.87
NFE	Inclusion level	−0.07	0.02	−3.27	0.001	−6.80
Starch	SpeciesGoat	−0.12	0.11	−1.03	0.303	−11.08
Starch	SpeciesSheep	−0.14	0.11	−1.19	0.232	−12.71
Starch	Clay_typeKaolinite	−0.12	0.12	−1.02	0.307	−11.13
Starch	Clay_typeMontmorillonite	0.01	0.00	1.91	0.056	0.71
Starch	Inclusion level	0.01	0.00	17.27	0.000	0.69

Note: Estimates represent regression coefficients (β) obtained from multilevel meta−regression models fitted using REML. β, Estimate (log−response ratio, main fixed effect); SE, standard errors; z, Wald z−statistic (β/SE), *p*−values, test of β = 0. OM, Organic matter; CP, Crude protein; EE, Ether extract; NDF, Neutral detergent fiber; ADF; Acid detergent fiber; DM, Dry matter; CF, Crude fiber; NFE, Nitrogen free extract. Relative percentage changes (%) for organic matter, crude protein, ether extract, neutral detergent fiber, acid detergent fiber, dry matter, crude fiber, nitrogen free extract and starch from multilevel random−effects meta−analysis, stratified by influencing factors (study design, clay type, dosage, ruminant species). Significant values were highlighted.

*Variability and its identification are written together as software came up with. Variability is put as red text for visual convenience.

3.3 Impacts of Clays on Health Indicators Across Studies

Table S10. Impacts of influencing factors other than control and treatment groups on ruminant health indicators.

Outcome	Influencing factors*	β	SE	z	*p*–values	Relative changes (%)
Albumin	SpeciesCattle	0.07	0.06	1.19	0.234	7.57
Albumin	SpeciesGoat	0.11	0.07	1.73	0.083	12.03
Albumin	SpeciesSheep	0.11	0.07	1.58	0.115	11.16
Albumin	Clay_typeMontmorillonite	0.02	0.04	0.39	0.696	1.62
Albumin	Clay_typeNano Montmorillonite	−0.03	0.07	−0.39	0.695	−2.78
Albumin	Clay_typeNano Zeolite	0.04	0.05	0.76	0.446	3.69
Albumin	Clay_typeOthers	0.004	0.03	0.13	0.900	0.43
Albumin	Clay_typeSepiolite	−0.01	0.07	−0.22	0.825	−1.43
Albumin	Clay_typeZeolite	0.01	0.02	0.45	0.653	0.93
Albumin	Inclusion level	−0.002	0.00	−1.04	0.297	−0.18
ALT	SpeciesGoat	0.16	0.12	1.35	0.178	17.74
ALT	SpeciesSheep	0.02	0.12	0.15	0.878	1.89
ALT	Clay_typeNano Montmorillonite	0.11	0.18	0.61	0.541	11.74
ALT	Clay_typeNano Zeolite	−0.08	0.42	−0.19	0.849	−7.62
ALT	Clay_typeOthers	0.05	0.12	0.45	0.654	5.49
ALT	Clay_typeSepiolite	0.04	0.15	0.24	0.808	3.83
ALT	Clay_typeZeolite	0.005	0.04	0.12	0.908	0.46
ALT	Inclusion level	0.001	0.01	0.16	0.870	0.11
AST	SpeciesGoat	−0.03	0.10	−0.28	0.782	−2.72
AST	SpeciesSheep	−0.17	0.11	−1.59	0.111	−15.66
AST	Clay_typeNano Montmorillonite	0.04	0.13	0.28	0.779	3.63
AST	Clay_typeNano Zeolite	0.16	0.39	0.41	0.685	17.04
AST	Clay_typeOthers	0.02	0.14	0.14	0.888	2.03
AST	Clay_typeSepiolite	0.22	0.16	1.38	0.169	25.10
AST	Clay_typeZeolite	0.06	0.04	1.70	0.089	6.72
AST	Inclusion level	0.01	0.01	1.04	0.299	0.53
Bilirubin	Clay_typeNano Montmorillonite	−3.80	0.49	−7.78	0.000	−97.77
Bilirubin	Inclusion level	−0.77	0.01	−78.11	0.000	−53.62
BUN	SpeciesSheep	0.08	0.10	0.77	0.444	7.80
BUN	Clay_typeNano Montmorillonite	−0.09	0.11	−0.81	0.418	−8.68
BUN	Inclusion level	−0.01	0.00	−1.39	0.164	−0.50
Calcium	SpeciesCattle	−0.10	0.06	−1.53	0.126	−9.33
Calcium	SpeciesGoat	−0.17	0.10	−1.63	0.102	−15.60
Calcium	Clay_typeMontmorillonite	0.02	0.08	0.26	0.792	2.01
Calcium	Clay_typeNano Zeolite	0.18	0.09	1.92	0.055	19.43
Calcium	Clay_typeOthers	−0.01	0.07	−0.09	0.929	−0.59

Calcium	Clay_typeZeolite	0.02	0.06	0.29	0.775	1.80
Calcium	Inclusion level	−0.004	0.01	0.32	0.749	0.17
Chloride	Clay_typeOthers	0.08	1.38	0.06	0.955	8.11
Chloride	Clay_typeZeolite	0.11	1.38	0.08	0.937	11.40
Chloride	Inclusion level	−0.004	0.06	−0.06	0.953	−0.35
CK	Clay_typeOthers	0.40	2.27	0.17	0.861	48.64
CK	Clay_typeZeolite	0.34	2.27	0.15	0.882	40.26
CK	Inclusion level	−0.01	0.10	−0.11	0.914	−1.06
Creatinine	SpeciesGoat	−0.04	0.04	−0.80	0.425	−3.51
Creatinine	SpeciesSheep	−0.14	0.07	−2.17	0.030	−13.32
Creatinine	Clay_typeMontmorillonite	−0.04	0.07	−0.49	0.625	−3.50
Creatinine	Clay_typeNano Montmorillonite	−0.16	0.12	−1.36	0.173	−14.75
Creatinine	Clay_typeOthers	0.01	0.03	0.21	0.833	0.53
Creatinine	Clay_typeZeolite	0.02	0.02	0.96	0.338	2.24
Creatinine	Inclusion level	−0.005	0.00	−0.92	0.356	−0.45
Globulin	SpeciesCattle	0.03	0.07	0.39	0.697	2.69
Globulin	SpeciesGoat	−0.17	0.06	−3.09	0.002	−15.68
Globulin	SpeciesSheep	−0.08	0.07	−1.18	0.240	−7.85
Globulin	Clay_typeMontmorillonite	−0.06	0.13	−0.47	0.641	−5.75
Globulin	Clay_typeNano Zeolite	−0.13	0.09	−1.35	0.178	−11.84
Globulin	Clay_typeOthers	−0.05	0.05	−0.90	0.366	−4.75
Globulin	Clay_typeZeolite	−0.10	0.01	−7.82	0.000	−9.24
Globulin	Inclusion level	−0.001	0.00	−0.29	0.775	−0.13
Glucose	SpeciesGoat	−0.18	0.11	−1.65	0.099	−16.41
Glucose	SpeciesSheep	−0.09	0.11	−0.83	0.409	−8.33
Glucose	Clay_typeMontmorillonite	0.10	0.03	3.44	0.001	10.34
Glucose	Clay_typeNano Montmorillonite	−0.20	0.12	−1.73	0.083	−18.18
Glucose	Clay_typeNano Zeolite	0.01	0.09	0.12	0.904	1.06
Glucose	Clay_typeOthers	−0.02	0.01	−2.07	0.039	−1.58
Glucose	Clay_typeSepiolite	−0.04	0.13	−0.32	0.746	−4.06
Glucose	Clay_typeZeolite	−0.09	0.01	−11.81	0.000	−8.84
Glucose	Inclusion level	0.002	0.00	0.83	0.405	0.17
HDL	SpeciesSheep	−0.03	0.34	−0.09	0.926	−3.08
HDL	Inclusion level	0.00	0.01	−0.02	0.986	−0.02
Sodium	SpeciesGoat	1.71	1.63	1.05	0.295	450.51
Sodium	Clay_typeMontmorillonite	0.02	0.02	1.39	0.163	2.19
Sodium	Inclusion level	−0.09	0.09	−1.00	0.316	−8.76
TC	SpeciesGoat	−0.04	0.08	−0.45	0.652	−3.52
TC	SpeciesSheep	−0.08	0.10	−0.81	0.416	−7.66
TC	Clay_typeMontmorillonite	−0.01	0.03	−0.26	0.794	−0.74
TC	Clay_typeNano Montmorillonite	−0.21	0.07	−3.17	0.002	−18.99
TC	Clay_typeNano Zeolite	−0.25	0.06	−4.37	0.000	−21.96
TC	Clay_typeSepiolite	−0.04	0.07	−0.61	0.540	−4.36
TC	Clay_typeZeolite	−0.01	0.08	−0.17	0.863	−1.42
TC	Inclusion level	−0.001	0.01	−0.10	0.917	−0.05

TG	SpeciesCattle	0.10	0.16	0.64	0.520	10.76
TG	SpeciesGoat	0.20	0.15	1.30	0.194	21.84
TG	SpeciesSheep	0.08	0.11	0.66	0.511	7.82
TG	Clay_typeMontmorillonite	0.10	0.17	0.57	0.569	10.11
TG	Clay_typeSepiolite	−0.17	0.11	−1.49	0.137	−15.24
TG	Clay_typeZeolite	−0.06	0.13	−0.47	0.635	−5.84
TG	Inclusion level	0.01	0.01	1.30	0.195	0.74
TP	SpeciesCattle	−0.02	0.05	−0.37	0.714	−1.92
TP	SpeciesGoat	−0.07	0.05	−1.34	0.181	−6.60
TP	SpeciesSheep	−0.01	0.05	−0.12	0.902	−0.60
TP	Clay_typeMontmorillonite	−0.01	0.05	−0.13	0.899	−0.69
TP	Clay_typeNano Montmorillonite	−0.04	0.10	−0.37	0.709	−3.61
TP	Clay_typeNano Zeolite	0.06	0.05	1.19	0.235	6.21
TP	Clay_typeOthers	−0.04	0.03	−1.31	0.191	−3.82
TP	Clay_typeSepiolite	0.02	0.06	0.34	0.733	1.95
TP	Clay_typeZeolite	−0.04	0.02	−1.86	0.062	−4.02
TP	Inclusion level	0.002	0.00	1.59	0.113	0.24
Urea	SpeciesGoat	−0.05	0.06	−0.82	0.412	−4.63
Urea	SpeciesSheep	−0.04	0.08	−0.49	0.623	−3.83
Urea	Clay_typeMontmorillonite	−0.01	0.14	−0.08	0.933	−1.18
Urea	Clay_typeNano Zeolite	0.15	0.09	1.76	0.078	16.20
Urea	Clay_typeOthers	0.01	0.03	0.22	0.828	0.74
Urea	Clay_typeSepiolite	−0.06	0.10	−0.61	0.540	−5.67
Urea	Clay_typeZeolite	0.02	0.02	1.24	0.217	2.24
Urea	Inclusion level	0.003	0.00	0.76	0.447	0.30

Note: Estimates represent regression coefficients (β) obtained from multilevel meta−regression models fitted using REML. β, Estimate (log−response ratio, main fixed effect); SE, standard errors; z, Wald z−statistic (β/SE), *p*−values, test of β = 0. ALT, Alanine Aminotransferase; AST, Aspartate Aminotransferase; BUN, Blood Urea Nitrogen; CK, Creatine kinase; HDL, High−Density Lipoprotein; TC, Total Cholesterol; TG, Triglycerides; TP, Total protein. Relative percentage changes (%) for albumin, ALT, AST, bilirubin, BUN, calcium, chloride, creatine kinase, creatine, globulin, glucose, HDL, sodium, TC, TG, total protein and urea from multilevel random−effects meta−analysis, stratified by influencing factors (study design, clay type, dosage, ruminant species). Significant values were highlighted.

*Variability and its identification are written together as software came up with. Variability is put as red text for visual convenience.

3.4 Functional role of Clays and Clay Minerals on Ruminants' Productivity

Table S11. Effects of factors other than control and treatment groups on ruminant health indicators and productivity.

**Outcome**	**Influencing factors***	**β**	**SE**	**z**	***p*–values**	**Relative changes (%)**
Weight gain	SpeciesCattle	−0.15	0.19	−0.78	0.434	−13.51
Weight gain	SpeciesSheep	−0.04	0.17	−0.23	0.817	−3.89
Weight gain	Clay_typeKaolinite	−0.02	0.33	−0.07	0.942	−2.37
Weight gain	Clay_typeMontmorillonite	−0.001	0.09	−0.01	0.989	−0.13
Weight gain	Clay_typeOthers	0.02	0.06	0.41	0.680	2.45
Weight gain	Clay_typeSepiolite	−0.09	0.10	−0.92	0.359	−8.85
Weight gain	Clay_typeZeolite	0.06	0.08	0.78	0.438	6.41
Weight gain	Clay_typeZeolite (60)	−0.03	0.10	−0.34	0.737	−3.15
Weight gain	Inclusion level	−0.004	0.00	−10.02	0.000	−0.42
Weight gain	Duration	0.00	0.00	0.14	0.890	0.02
Milk yield	SpeciesGoat	0.02	0.09	0.21	0.832	2.03
Milk yield	Clay_typeMontmorillonite	−0.004	0.03	−0.12	0.902	−0.42
Milk yield	Clay_typeNano Zeolite	0.26	0.15	1.80	0.072	29.88
Milk yield	Clay_typeOthers	−0.06	0.07	−0.81	0.420	−5.44
Milk yield	Clay_typeZeolite	−0.11	0.03	−3.42	0.001	−10.62
Milk yield	Inclusion level	0.001	0.01	0.19	0.853	0.15
Milk yield	Duration	−0.001	0.00	−0.61	0.539	−0.06
Fat yield	SpeciesGoat	−0.11	0.08	−1.38	0.169	−9.99
Fat yield	Clay_typeMontmorillonite	−0.005	0.02	−0.20	0.842	−0.48
Fat yield	Clay_typeNano Zeolite	0.03	0.15	0.17	0.867	2.55
Fat yield	Clay_typeOthers	−0.10	0.00	−52.47	0.000	−9.56
Fat yield	Clay_typeZeolite	−0.16	0.00	−82.47	0.000	−14.77
Fat yield	Inclusion level	−0.01	0.01	−0.74	0.457	−0.56
Fat yield	Duration	0.002	0.00	2.29	0.022	0.21
Protein yield	SpeciesGoat	0.01	0.14	0.11	0.916	1.50
Protein yield	Clay_typeMontmorillonite	0.10	0.04	2.85	0.004	10.79
Protein yield	Clay_typeNano Zeolite	−0.03	0.22	−0.15	0.884	−3.20
Protein yield	Clay_typeOthers	0.11	0.20	0.55	0.580	11.96
Protein yield	Clay_typeZeolite	−0.17	0.05	−3.45	0.001	−15.27
Protein yield	Inclusion level	−0.004	0.01	−0.35	0.728	−0.42
Protein yield	Duration	0.003	0.00	1.05	0.294	0.32
Lactose yield	SpeciesGoat	0.01	0.06	0.15	0.878	0.98
Lactose yield	Clay_typeMontmorillonite	−0.05	0.04	−1.37	0.171	−5.12
Lactose yield	Clay_typeNano Zeolite	−0.03	0.20	−0.15	0.878	−3.07
Lactose yield	Clay_typeOthers	0.03	0.14	0.23	0.822	3.28
Lactose yield	Clay_typeZeolite	−0.14	0.04	−3.42	0.001	−13.49
Lactose yield	Inclusion level	−0.01	0.01	−0.52	0.603	−0.57
Lactose yield	Duration	0.003	0.00	1.14	0.255	0.27
Fat content	SpeciesGoat	−0.09	0.11	−0.84	0.400	−9.04
Fat content	Clay_typeMontmorillonite	−0.01	0.02	−0.37	0.709	−0.65

Fat content	Clay_typeNano Zeolite	0.31	0.09	3.65	0.000	36.99
Fat content	Clay_typeOthers	−0.07	0.07	−0.99	0.322	−7.06
Fat content	Clay_typeZeolite	−0.18	0.04	−4.71	0.000	−16.35
Fat content	Inclusion level	0.02	0.00	6.76	0.000	1.89
Fat content	Duration	−0.002	0.00	−1.91	0.056	−0.18
Protein content	SpeciesGoat	−0.01	0.08	−0.07	0.948	−0.53
Protein content	Clay_typeMontmorillonite	0.10	0.00	21.14	0.000	10.47
Protein content	Clay_typeNano Zeolite	0.21	0.03	7.62	0.000	23.45
Protein content	Clay_typeOthers	−0.03	0.02	−1.50	0.134	−2.93
Protein content	Clay_typeZeolite	−0.07	0.01	−4.50	0.000	−6.34
Protein content	Inclusion level	0.01	0.00	14.04	0.000	1.46
Protein content	Duration	−0.001	0.00	−1.39	0.165	−0.09
Lactose content	SpeciesGoat	0.08	0.03	3.10	0.002	8.26
Lactose content	Clay_typeMontmorillonite	−0.06	0.01	−4.78	0.000	−5.50
Lactose content	Clay_typeNano Zeolite	−0.13	0.02	−6.30	0.000	−12.43
Lactose content	Clay_typeOthers	−0.02	0.01	−1.11	0.266	−1.57
Lactose content	Clay_typeZeolite	−0.02	0.01	−2.13	0.033	−2.28
Lactose content	Inclusion level	−0.005	0.00	−7.57	0.000	−0.48
Lactose content	Duration	0.00	0.00	2.11	0.035	0.05

Note: Estimates represent regression coefficients (β) obtained from multilevel meta−regression models fitted using REML. β, Estimate (log−response ratio, main fixed effect); SE, standard errors; z, Wald z−statistic (β/SE), *p*−values, test of β = 0. Relative percentage changes (%) for weight gain, milk yield, fat yield, protein yield, lactose yield, fat content, protein content and lactose content from multilevel random−effects meta−analysis, stratified by influencing factors (study design, clay type, dosage, ruminant species). Significant values were highlighted.

*Variability and its identification are written together as software came up with. Variability is put as red text for visual convenience.

3.6 Certainty of Evidence Across Studies

Table S12. Assessment of certainty of evidence using GRADE domains

Outcome domain	Key outcomes considered	Risk of bias	Inconsistency	Indirectness	Imprecision	Publication bias	Overall certainty
Rumen fermentation and methane mitigation	CH_4, NH_3–N, propionate, total VFA, pH	Not serious	Serious	Not serious	Not serious	Serious	Low
Nutrient digestibility	DMD, OMD, NDFD, ADFD, CPD	Not serious	Very serious	Not serious	Not serious	Serious	Low
Animal health	BUN and blood metabolites	Not serious	Serious	Not serious	Serious	Not serious	Low
Productive performance	Weight gain, milk yield, fat yield, lactose yield	Not serious	Very serious	Not serious	Not serious	Serious	Low

Note: Certainty of evidence was assessed using the GRADE domains of risk of bias, inconsistency, indirectness, imprecision, and publication bias, adapted for animal nutrition meta−analysis. Risk of bias was judged as not serious because most studies met the predefined quality criteria (Table S2). Inconsistency was evaluated using I^2 values (<50%, not serious; 50–75%, serious; >75%, very serious), while publication bias was assessed using Egger’s regression tests and trim−and−fill analyses. Imprecision was considered serious when evidence was limited by a small number of studies and/or wide confidence intervals. Overall certainty ratings were assigned based on the combined assessment of these domains.

References

1. Al Adawi, S.A.; El-Zaiat, H.M.; Morsy, A.S.; Soltan, Y.A. Lactation Performance and Rumen Fermentation in Dairy Cows Fed a Diet Supplemented with Monensin or Gum Arabic-Nano Montmorillonite Compost. *Animals (Basel)* **2024**, *14*, 1649, doi:10.3390/ani14111649.
2. Alrez, M.; Jammoul, A.; Al-Rahmoun, W.; Al-Omar, Y. The assessment of zeolite addition to diets with or without urea on some productive and physiological indicators in Awassi lambs. *BMC Veterinary Research* **2024**, *20*, 386.
3. Antonelo, D.S.; Lancaster, N.A.; Melnichenko, S.; Muegge, C.R.; Schoonmaker, J.P. Effects of clay on toxin binding capacity, ruminal fermentation, diet digestibility, and growth of steers fed high-concentrate diets. *J Anim Sci* **2017**, *95*, 4658-4667, doi:10.2527/jas2017.1473.
4. Azadbakht, S.; Khadem, A.; Norouzian, M. Bentonite supplementation can improve performance and fermentation parameters of chronic lead-exposed lambs. *Environmental Science and Pollution Research* **2017**, *24*, 5426-5430.
5. Damato, A.; Vanzani, P.; Giannuzzi, D.; Giaretta, E.; Novelli, E.; Vianello, F.; Tagliapietra, F.; Zennaro, L. Bentonite does not affect in vitro ruminal gross fermentations but could modify ruminal metabolome and mineral content. A proof of concept. *Res Vet Sci* **2022**, *144*, 78-81, doi:10.1016/j.rvsc.2022.01.012.
6. El-Nile, A.; Elazab, M.; El-Zaiat, H.; El-Azrak, K.E.; Elkomy, A.; Sallam, S.; Soltan, Y. In Vitro and In Vivo Assessment of Dietary Supplementation of Both Natural or Nano-Zeolite in Goat Diets: Effects on Ruminal Fermentation and Nutrients Digestibility. *Animals (Basel)* **2021**, *11*, 2215, doi:10.3390/ani11082215.
7. El-Nile, A.E.; Elazab, M.A.; Soltan, Y.A.; Elkomy, A.E.; El-Zaiat, H.M.; Sallam, S.M.A.; El-Azrak, K.E.-D. Nano and natural zeolite feed supplements for dairy goats: Feed intake, ruminal fermentation, blood metabolites, and milk yield and fatty acids profile. *Animal Feed Science and Technology* **2023**, *295*, 115522, doi:10.1016/j.anifeedsci.2022.115522.
8. Aladdin Helmy, S.; Mahrous Ebeid, H.; Ahmed Hanafy, M.; Mohamed Mahmoud, A.E.; Roshdy Ali El-Tanany, R. Rumen Parameters, Nutrients Digestibility and Milk Production of Lactating Boer Goats Fed Diets Containing Clay Minerals. *Pak J Biol Sci* **2022**, *25*, 755-764, doi:10.3923/pjbs.2022.755.764.
9. Islam, M.R.; Naidu, R.; Biswas, B. Natural kaolin minerals with varying iron contents reduced cattle's enteric methane generation in vitro. *Applied Geochemistry* **2025**, *189*, 106459, doi:10.1016/j.apgeochem.2025.106459.
10. Jiang, Y.; Ogunade, I.M.; Kim, D.H.; Li, X.; Pech-Cervantes, A.A.; Arriola, K.G.; Oliveira, A.S.; Driver, J.P.; Ferraretto, L.F.; Staples, C.R.; et al. Effect of adding clay with or without a Saccharomyces cerevisiae fermentation product on the health and performance of lactating dairy cows challenged with dietary aflatoxin B(1). *J Dairy Sci* **2018**, *101*, 3008-3020, doi:10.3168/jds.2017-13678.
11. Myers, C.A.; de Haro Marti, M.E.; Chahine, M.; Chibisa, G.E. Feeding or pen surface application of clinoptilolite with different particle sizes: impact on nitrogen utilization and manure ammonia emissions in feedlot cattle. *Journal of Animal Science* **2024**, *102*, skae230.
12. Othman, A.; Taha, T.; Al-Shafii, M.; Deskoy, T.; Ratib, M.; Youssef, M.; Mohammed, E.; Soltan, Y.A. Use of red seaweed phytochemicals-zeolite nanocomposite as a feed additive to reduce ruminal methane emissions in vitro. *Tropical Animal Health and Production* **2025**, *57*, 266.

13. Urías-Estrada, J.D.; Estrada-Angulo, A.; Castro-Pérez, B.I.; Ponce-Barraza, E.; Arteaga-Wences, Y.J.; Ramos-Méndez, J.L.; Barreras, A.; Valdés-García, Y.S.; Plascencia, A. Impact of presence of zeolite in diets for lambs supplemented with zilpaterol hydrochloride: Growth performance and dietary energetics. *Chilean journal of agricultural research* **2023**, *83*, 768-776.
14. Zeltwanger, J.M.; Bailey, E.A.; Petzel, E.A.; Nelson, B.M.; Brake, D.W.; Leonhard, J.T.; Canterbury, L.G.; Hergenreder, J.E. Ability of an enhanced zeolite-based flow agent to mitigate the effects of ergot-like alkaloids consumed by beef cattle. *Applied Animal Science* **2024**, *40*, 504-510.
15. Amanzougarene, Z.; Fondevila, M. Rumen Fermentation of Feed Mixtures Supplemented with Clay Minerals in a Semicontinuous In Vitro System. *Animals (Basel)* **2022**, *12*, 345, doi:10.3390/ani12030345.
16. Schlattl, M.; Buffler, M.; Windisch, W. Clay Minerals Affect the Solubility of Zn and Other Bivalent Cations in the Digestive Tract of Ruminants In Vitro. *Animals (Basel)* **2021**, *11*, 877, doi:10.3390/ani11030877.
17. Abo-Sherif, S.; Sallam, S.; Allam, A.M.; El-Adawy, M.; Soltan, Y. In Vitro Evaluation of Ruminal Fermentation and Methane Production in Response to the Addition of Modified Nano-Bentonite with or Without Saccharomyces cerevisiae to a Forage-Based Diet. *Animals* **2025**, *15*, 2081.
18. Pikhtirova, A.; Pecka-Kielb, E.; Kroliczewska, B.; Zachwieja, A.; Kroliczewski, J.; Kupczynski, R. The Effect of Saponite Clay on Ruminal Fermentation Parameters during In Vitro Studies. *Animals (Basel)* **2024**, *14*, 738, doi:10.3390/ani14050738.
19. Chen, L.; Li, F.; Ma, Z.; Wang, A.; Kang, Y.; Liu, B.; Xu, H.; Li, J.; Wang, X.; Li, K. Improving feed intake and rumen fermentation in lambs using mixed-dimensional attapulgite clay to adsorb naturally occurring mycotoxins. *J Anim Sci* **2024**, *102*, skae080, doi:10.1093/jas/skae080.
20. Sulzberger, S.A.; Kalebich, C.C.; Melnichenko, S.; Cardoso, F.C. Effects of clay after a grain challenge on milk composition and on ruminal, blood, and fecal pH in Holstein cows. *J Dairy Sci* **2016**, *99*, 8028-8040, doi:10.3168/jds.2016-11030.
21. Ortiz, J.; Montano, M.; Plascencia, A.; Salinas, J.; Torrentera, N.; Zinn, R.A. Influence of Kaolinite Clay Supplementation on Growth Performance and Digestive Function in Finishing Calf-fed Holstein Steers. *Asian-Australas J Anim Sci* **2016**, *29*, 1569-1575, doi:10.5713/ajas.16.0162.
22. Gouda, G.A.; Khattab, H.M.; Abdel-Wahhab, M.A.; Abo El-Nor, S.A.; El-Sayed, H.M.; Kholif, S.M. Clay minerals as sorbents for mycotoxins in lactating goat's diets: Intake, digestibility, blood chemistry, ruminal fermentation, milk yield and composition, and milk aflatoxin M1 content. *Small Ruminant Research* **2019**, *175*, 15-22, doi:10.1016/j.smallrumres.2019.04.003.
23. Soltan, Y.A.; Morsy, A.S.; Hashem, N.M.; Elazab, M.A.I.; Sultan, M.A.; El-Nile, A.; Marey, H.N.; El Lail, G.A.; El-Desoky, N.; Hosny, N.S.; et al. Potential of montmorillonite modified by an organosulfur surfactant for reducing aflatoxin B1 toxicity and ruminal methanogenesis in vitro. *BMC Vet Res* **2022**, *18*, 387, doi:10.1186/s12917-022-03476-1.
24. Hussein, Y.; Abdo, S.; Hassan, E.; Farghali, M.; Farghaly, M. Effect of clay minerals supplementation on nutrient digestibility, blood parameters, and performance of buffalo calves. *Archives of Agriculture Sciences Journal* **2023**, *0*, 95-106, doi:10.21608/aasj.2023.205721.1148.
25. Fabino Neto, R.; Ribeiro, M.D.; José da Silva, C.; Cabral Filho, S.L.S.; Oliveira, R.V.; Brainer, M.M.d.A.; Zacaroni, O.d.F.; Diogo, J.M.d.S.; Arnhold, E.; Oliveira,

R.S. The Effect of Clayey Micromineral Compounds in Lamb Feed on Health, Intake, Performance, and Carcass and Meat Quality Parameters. *Ruminants* **2025**, *5*, 52.
26. Tate, K.; Yuan, G.; Theng, B.; Churchman, G.; Singh, J.; Berben, P. Can geophagy mitigate enteric methane emissions from cattle? *Journal of Prelimary Research* **2015**, *2*, 1-8, doi:10.1111/JPR.V2I1.103.
27. Gallo, A.; Rocchetti, G.; Piccioli Cappelli, F.; Pavone, S.; Mulazzi, A.; van Kuijk, S.; Han, Y.; Trevisi, E. Effect of a commercial bentonite clay (smectite clay) on dairy cows fed aflatoxin-contaminated feed. *Dairy* **2020**, *1*, 135-153, doi:10.3390/dairy1020009.
28. Mehany, A.; Shams, A.S.E. Effect of toxin binder on productive performance of lactating Friesian cows. *Journal of Animal and Poultry Production* **2019**, *10*, 405-413.
29. Burçak, E.; Yalçın, S. Effects of dietary sepiolite usage on performance, carcass characteristics, blood parameters and rumen fluid metabolites in Merino cross breed lambs. *Applied Clay Science* **2018**, *163*, 291-298.
30. Razavi, S.A.; Pourjafar, M.; Hajimohammadi, A.; Valizadeh, R.; Naserian, A.A.; Laven, R.; Mueller, K.R. Effects of dietary supplementation of bentonite and yeast cell wall on serum blood urea nitrogen, triglyceride, alkaline phosphatase, and calcium in high-producing dairy cattle during the transition period. *Comparative Clinical Pathology* **2019**, *28*, 419-425.
31. Kihal, A.; Marquès, C.; Rodríguez-Prado, M.; Jose-Cunilleras, E.; Calsamiglia, S. Effect of diet supplementation with the mycotoxin binder montmorillonite on the bioavailability of vitamins in dairy cows. *Toxins* **2022**, *14*, 26.
32. Biswas, A.A.; Lee, S.S.; Mamuad, L.L.; Kim, S.H.; Choi, Y.J.; Lee, C.; Lee, K.; Bae, G.S.; Lee, S.S. Effects of illite supplementation on in vitro and in vivo rumen fermentation, microbial population and methane emission of Hanwoo steers fed high concentrate diets. *Anim Sci J* **2018**, *89*, 114-121, doi:10.1111/asj.12913.
33. Zhang, W.; Zhang, L.; Jiang, X.; Liu, X.; Li, Y.; Zhang, Y. Enhanced adsorption removal of aflatoxin B1, zearalenone and deoxynivalenol from dairy cow rumen fluid by modified nano-montmorillonite and evaluation of its mechanism. *Animal Feed Science and Technology* **2020**, *259*, 114366.
34. Maki, C.R.; Thomas, A.D.; Elmore, S.E.; Romoser, A.A.; Harvey, R.B.; Ramirez-Ramirez, H.A.; Phillips, T.D. Effects of calcium montmorillonite clay and aflatoxin exposure on dry matter intake, milk production, and milk composition. *J Dairy Sci* **2016**, *99*, 1039-1046, doi:10.3168/jds.2015-10242.
35. Castro-Pérez, B.I.; Rodríguez-Vázquez, J.; Estrada-Angulo, A.; Ríos-Rincón, F.G.; Urías-Estrada, J.D.; Ponce-Barraza, E.; Barreras, A.; Plascencia, A. Effect of particle size of zeolite clay included in high-energy diets for feedlot lambs: Growth performance, dietary energy, carcass traits, and visceral mass. *Journal of Advanced Veterinary and Animal Research* **2023**, *10*, 500.
36. Maki, C.; Haney, S.; Wang, M.; Ward, S.; Rude, B.; Bailey, R.; Harvey, R.; Phillips, T. Calcium montmorillonite clay for the reduction of aflatoxin residues in milk and dairy products. *J. Dairy Vet. Sci* **2017**, *2*, 1-8.
37. Aydın, Ö.D.; Merhan, O.; Yıldız, G. The effect of sodium bentonite on growth performance and some blood parameters in post-weaning Tuj breed lambs. *Ankara Üniversitesi Veteriner Fakültesi Dergisi* **2020**, *67*, 235-241.
38. Ismail, A.A.; Ibrahim, M.A.; Abdl-Rahman, M.A.; Sawiress, F.A. Comparative Physiological Assessment of Three Natural Feed Additives on In-vitro Rumen Fermentation Efficiency. *Veterinary Medical Journal (Giza)* **2025**, *71*, 69-83.
39. Roque-Jiménez, J.; Pinos-Rodríguez, J.; Rojo-Rub, R.; Mendoza, G.; Vazquez, A.; De Jesus, J.C.; Lee-Rangel, H. Effect of natural zeolite on live weight changes, ruminal

fermentation and nitrogen metabolism of ewe lambs. *South African Journal of Animal Science* **2018**, *48*.
40. Helmy, S.A.; Ebeid, H.M.; Hanafy, M.A.; Mahmoud, A.E.M.; El-Tanany, R.R.A. Rumen Parameters, Nutrients Digestibility and Milk Production of Lactating Boer Goats Fed Diets Containing Clay Minerals. *Pakistan journal of biological sciences: PJBS* **2022**, *25*, 755-764.
